# Knowledge and awareness of oral cancer among adults in North-Western Italy: A cross-sectional questionnaire-based survey in community pharmacies

**DOI:** 10.1371/journal.pone.0354509

**Published:** 2026-07-24

**Authors:** Francesca Baratta, Federica Romano, Paola Brusa, Paolo Giacomo Arduino

**Affiliations:** 1 Department of Drug Science and Technology, University of Turin, Turin, Italy; 2 CIR Dental School, Department of Surgical Sciences, University of Turin, Turin, Italy; University of the Republic Uruguay: Universidad de la Republica Uruguay, URUGUAY

## Abstract

Early detection is a key determinant of prognosis in oral squamous cell carcinoma (OSCC); however, delayed diagnosis remains common. Insufficient public awareness and knowledge of OSCC have been identified as important barriers to timely diagnosis. Therefore, the aim of this observational study is to assess knowledge of OSCC and its risk factors, and to identify possible differences in awareness and information sources according to demographic and subject-related factors. A cross-sectional survey was conducted between July 2023 and June 2024 in community pharmacies in North-Western Italy. Adult customers completed a structured questionnaire administered by trained interviewers. Associations were evaluated using chi-square tests and multivariable logistic regression analyses. A total of 914 participants were consecutively included (mean age 54.1 ± 17.0 years). Overall, 70.4% were aware of OSCC, mostly from family/friends (51.8%) and media (43.2%). Smoking was widely recognized as a risk factor (97.9%), while alcohol consumption and sunlight exposure were less known (63.9% and 18.6%, respectively). Awareness was significantly influenced by gender, higher educational level, age and smoking status. Most respondents (92.2%) expressed a need for further information, with community pharmacies identified as the preferred source (67.3%). The present findings highlight the need for OSCC awareness campaigns in North-Western Italy and suggest that community pharmacies may represent a valuable setting for delivering such interventions.

## Introduction

Oral squamous cell carcinoma (OSCC) is a major public health concern and represents the most frequent malignant tumour of the oral cavity. Globally, it accounts for more than 300,000 newly diagnosed cases each year and is associated with a substantial mortality burden, representing approximately 2% of all cancer-related deaths worldwide [1]. In Italy alone, nearly 10,000 new cases were reported in 2018, with a marked predominance in the male population [2].

Traditionally, OSCC has been considered a disease primarily affecting men over the age of 50; however, recent epidemiological evidence indicates a growing incidence among women and younger individuals, highlighting a shift in the demographic profile of affected patients. Despite advances in clinical management, diagnostic techniques, and therapeutic approaches, survival outcomes for OSCC have shown limited improvement over recent decades. The overall five-year survival rate remains around 50%, largely depending on the stage at which the disease is diagnosed [3–5].

Because early-stage lesions are often clinically visible within the oral cavity, timely diagnosis may substantially improve prognosis and treatment outcomes. Prompt identification of these lesions can result in better outcomes and improved quality of life [6]. Nevertheless, many patients are still diagnosed at advanced stages, when treatment becomes more complex and outcomes are less favourable. Beyond exposure to established risk factors such as tobacco and alcohol consumption, delayed diagnosis has been strongly associated with insufficient recognition of early signs and symptoms of the disease by the general population [7]. Lack of awareness may result in postponed healthcare-seeking behaviour, ultimately reducing the effectiveness of early intervention strategies. Late diagnosis of OSCC not only negatively affects survival but also has significant consequences for patients’ quality of life. Advanced disease often requires extensive surgical procedures, adjuvant therapies, and prolonged hospitalization, leading to functional, aesthetic, and psychological impairments, as well as increased healthcare costs [8–10]. Previous studies and systematic reviews have consistently identified limited population knowledge and awareness as major contributors to diagnostic delay in OSCC [11]. A recent Italian survey-based study has reported that public awareness of OSCC remains limited and is mainly influenced by gender and educational level [12]. While smoking is generally recognized as a major risk factor, knowledge of other established risk factors is less widespread, particularly among individuals with lower education. Additionally, the presence of misconceptions regarding OSCC aetiology highlights the persistence of misinformation within the general population.

Despite existing evidence, data on public knowledge and awareness of OSCC remain limited and fragmented, particularly at the regional level [12]. Moreover, differences in awareness according to demographic and subject-related factors are not yet fully understood, and misinformation continues to represent a relevant public health issue [13]. In this context, assessing population knowledge and awareness of OSCC is essential to identify informational gaps and to support the development of targeted preventive and educational interventions [14].

Community pharmacies, owing to their widespread geographic distribution, accessibility and extended opening hours, are well positioned to play a significant role in preventive activities across a broad range of conditions [15,16]. Consistently, several campaigns have successfully engaged Italian community pharmacies in primary, secondary and tertiary prevention initiatives [17–21]. These findings suggest a potential role for community pharmacies also in the context of OSCC awareness and prevention [22]. Therefore, this study was designed as a survey with two main objectives: first, to assess knowledge and awareness of OSCC among adults and to explore potential differences according to demographic and participant-related characteristics; second, to gather information on the potential role of community pharmacies in this field.

## Materials and methods

### Study design and survey development

A cross-sectional study was conducted among customers attending community pharmacies across the Piedmont Region, located in North-West Italy, between 24 July 2023 and 28 June 2024. The study was reported in accordance to the STROBE (Strengthening the Reporting of Observational Studies in Epidemiology) statement [23] and the CROSS (Checklist for Reporting Of Survey Studies) recommendations [24].

Data were collected through face-to-face interviews using a structured questionnaire administered by trained researchers from the University of Turin. The project was presented to customers with the support of the pharmacists involved in the participating pharmacies. All interviewers completed a standardized training programme to ensure consistency in questionnaire administration and to minimize interviewer-related bias.

The questionnaire was specifically developed for this study, using constructs and items adapted from previously published and validated survey assessing awareness and knowledge of OSCC and cancer-related topics [12,25]. The adaptation process involved selecting relevant items, followed by review and refinement by the research team to ensure alignment with the study objectives and appropriateness for the target population. A pilot test was conducted among 20 pharmacy customers to evaluate readability, comprehensibility and acceptability of the questionnaire items. Minor modifications to wording and item phrasing were introduced based on participant feedback before the start of the main survey. Content validity was assessed through expert review by five university professors using the content validity ratio (CVR) and content validity index (CVI) [26]. All items met the minimum acceptable CVR threshold, and the overall CVI was 0.92, indicating excellent content validity and expert agreement regarding item relevance and clarity. Because the questionnaire primarily comprised factual and knowledge-based items rather than scales measuring latent psychometric constructs, internal consistency measures such as Cronbach’s alpha were not considered appropriate. A full psychometric validation process, including construct validity, criterion validity, and test–retest reliability, was not performed, and should be considered when interpreting the results.

The final questionnaire consisted of 15 items, including two open-ended questions, one dichotomous question, and twelve with multiple-choice options. It was organized into three main sections. The first section focused on the sociodemographic background of the participants (age, gender, educational level, frequency of dental check-ups, and smoking habits). The second section explored participants’ awareness and knowledge of OSCC, including sources of information, risk factors, tumour characteristics, survival, early diagnosis, age of onset, signs and symptoms, and appropriate healthcare professionals to consult in case of suspicion. OSCC awareness was treated as a binary variable, with participants classified as aware if they reported having previously heard of OSCC [25]. Finally, the third section of the questionnaire explored perceptions regarding healthcare professionals involved in OSCC management and preferred channels for disseminating information. The survey questionnaire is available as Supporting Information (S1 File).

### Participants and recruitment

Men and women, aged 18 years or older, who were able to understand and speak Italian were eligible to participate. Individuals with evident cognitive impairment or reporting a previous diagnosis of OSCC were excluded. These eligibility criteria were intended to ensure adequate comprehension of the questionnaire, while capturing a broad range of demographic and educational backgrounds.

Participation was voluntary and no incentives were offered. After completion of the questionnaire, all participants received an educational leaflet on OSCC, including information on risk factors, symptoms, and preventive measures. The leaflet was developed in accordance with recommendations from the Italian Society of Oral Pathology and Medicine.

### Ethical considerations

The study protocol was approved by the Bioethics Committee of the University of Turin (protocol no. 0494208, 18 July 2023). All participants received written information regarding the study objectives and procedures and provided written informed consent before participation. Participation was anonymous, no personally identifiable information was collected, and data confidentiality was maintained throughout the study.

### Sample size and sampling strategy

The minimum sample size was calculated to estimate the prevalence of OSCC awareness among customers attending community pharmacies. Assuming an expected prevalence of 69% [12], a 95% confidence level (CI), and a precision of 4%, a minimum sample size of 514 participants was required under simple random sampling assumptions. To account for clustering at the pharmacy level, the resulting final sample size was increased to approximately 910 participants. An intra-cluster correlation coefficient of 0.03 was assumed, consistent with values reported in similar community-based surveys and reflecting the expected low within-pharmacy correlation, where no shared educational intervention or clinical exposure exists.

To implement the sampling strategy, 115 community pharmacies were included using a non-random convenience sampling approach to ensure geographical representation across provinces of the Piedmont Region and inclusion of both urban-rural settings, assuming an approximately equal cluster size for each site. Within each participating pharmacy, consecutive eligible customers were invited to participate until the recruitment target was reached, with minor variations allowed based on feasibility.

### Statistical analysis

Statistical analyses were performed using SPSS (version 28.0, IBM Corp, Armonk, New York, USA). Quantitative variables were summarized as mean ± standard deviation (SD), and categorical variables were reported as frequencies and percentages. Associations between categorical variables were assessed using the chi-square test.

No missing data were recorded for the variables included in the analyses; therefore, complete-case analysis was performed. Although some non-response occurred during data collection, the response rate was high, and the planned sample size was achieved. Consequently, no statistical adjustment for non-response was applied.

Multivariable logistic regression analyses were conducted to identify independent predictors of OSCC awareness and knowledge of OSCC risk factors. Age (< 60 years versus ≥ 60 years), gender, educational level (categorized into three levels: low, including primary and secondary school; intermediate, including high school; and high, including university or above), smoking habits (current cigarette or e-cigarette users versus non- and former smokers), and dental attendance (regular versus irregular) were included in the models based on prior evidence. Although a backward stepwise elimination procedure (removing variables with p > 0.05) was initially considered, all variables were retained in the final model to provide fully adjusted estimates. Model fit was assessed using the Hosmer–Lemeshow goodness-of-fit test. Results are presented as adjusted odds ratio (AOR) with 95% CI, and statistical significance was set at p < 0.05.

## Results

### Descriptive data

A total of 961 eligible participants were approached consecutively; 914 agreed to participate and completed the survey, resulting in a response rate of 95.1%.

The sociodemographic characteristics of the participants are summarized in Table 1. The mean age was 54.1 ± 17.0 years (range: 18–92 years). More than half of the participants were female (62.4%), and the majority had at least a high school education. Approximately one-third were current smokers (28.5%), and 36.0% reported visiting the dentist only occasionally.

**Table 1:** Socio-demographic characteristics of participants (n = 914).

Variable	Category	Percentage (%)
Gender	Female	62.4
	Male	37.6
Age	18-39 years	22.2
	40-59 years	36.3
	≥ 60 years	41.5
Smokers	No	71.5
	Cigarettes	20.8
	E-cigarettes	7.7
Education	Low	21.3
	Moderate	42.2
	High	36.5
Dental attendance	Occasional	36.0
	Once a year	33.4
	Twice a year	30.6

### Knowledge about OSCC

#### Risk factors, warning signs/symptoms and source of information.

The results of the questionnaires are presented in Tables 2 and 3. Overall, 70.4% of participants reported being aware of the existence of OSCC. Information about the disease was obtained mainly from family/friends (51.8%) and media (43.2%), followed by healthcare professionals such as physicians and pharmacists (27.3%). Internet was the primary source for younger participants (p < 0.001), while those over 40 favoured interpersonal sources, including pharmacists (p = 0.002) and friends (p < 0.001). Higher educated subjects also predominantly identified general practitioners and pharmacists as their main sources of information (both p < 0.001).

**Table 2:** Frequency distribution of awareness of oral cancer, risk factors and source of information among participants.

Variable	Category	Percentage (%)
**Awareness of malignancy**	Oral cancer	88.5
	Lung cancer	96.3
	Liver cancer	85.8
	Uterine fibroma	35.9
	Lipoma	13.7
	Angioma	21.0
	None of the above	0.8
**Age group more often affected by oral cancer**	20–39 years	14.6
	40–59 years	73.1
	60–90 years	12.3
	> 90 years	0.1
**Oral cancer aggressiveness**	High	64.1
	Moderate	2.8
	I don’t know	33.0
**Oral cancer risk factors**	Smoking habits	97.9
	Drinking alcohol regularly	63.9
	Chronic irritation	52.7
	Poor oral hygiene	52.4
	Sun exposure	18.6
Source of information*	General practitioner	17.7
	Pharmacist	9.6
	Family/friends	51.8
	Internet	21.9
	Television	21.3
	Other	11.0
**Healthcare professional to consult for suspected oral cancer**	General practitioner	49.0
	Dentist	46.3
	Oncologist	41.1
	Otolaryngologist	47.6
	Pharmacist	5.6
	Dermatologist	5.0

* Percentages calculated among participants aware of oral cancer (n = 643).

**Table 3:** Frequency distribution of knowledge of oral cancer signs/symptoms among participants (n = 914).

Variable	Category	Percentage (%)
**Symptoms**	Insomnia	6.8
	Dizziness	16.7
	Numbness of the lips	65.1
**Signs**	Speech impairment	39.1
	Difficulty swallowing	67.6
	Loose teeth	39.1
	Blackened teeth	25.2
	Cervical lymphadenopathy	59.5
	Oral nodules	76.5
	Red or white oral patches	63.6
	Unexplained weight loss	53.0
	Hair loss	10.3
	Persistent cold	9.0
	Non-healing ulcers in the mouth	82.5
	Difficulty chewing	56.5

Most of the respondents correctly recognized OSCC as a malignant tumour (88.5%), with similar levels of awareness for other common malignancies, such as liver (85.8%) and lung cancer (96.3%). However, approximately one-third of participants were unaware of the aggressiveness of oral cancer. Furthermore, 73.1% identified adults aged 40–59 years as the age group most frequently affected.

Smoking was recognized as a major risk factor for OSCC by nearly all respondents (97.9%), while approximately two-thirds acknowledged alcohol consumption as an additional risk factor (63.9%). More than half of participants attributed risk to chronic trauma/irritation (52.7%) and poor oral hygiene habits (52.4%). In contrast, sunlight exposure was acknowledged by only 18.6% of respondents.

Regarding clinical manifestations (Table 3), non-healing oral ulcerations were most frequently identified as a warning sign of OSCC (82.5%), followed by oral nodules (76.5%), swallowing difficulties (67.6%), and numbness of the lip (65.1%). Red or white oral patches were also commonly associated with a possible oral malignancy (63.6%). Conversely, only a minority of participants identified symptoms such as insomnia, dizziness, and hair loss as suggestive of OSCC.

When asked which healthcare professional they would consult in the event of suspected oral cancer (Table 2), the most frequently selected option was the general practitioner (49.0%), followed closely by the otolaryngologist (47.6%), dentist (46.3%), and oncologist (41.1%). Only a small proportion indicated they would seek care from a pharmacist (5.6%) or a dermatologist (5.0%).

### Variables affecting knowledge about oral cancer and its risk factors

Table 4 presents the predictors of OSCC awareness. In the bivariate analysis, awareness of OSCC was significantly associated with gender (p < 0.001), age (p = 0.032), educational level (p < 0.001), smoking status (p = 0.028), and regular dental attendance (p = 0.020). In the multivariable logistic regression analysis, female participants were significantly more likely to be aware of OSCC than male participants (AOR = 1.67, 95% CI: 1.23–2.26; p = 0.001). Cigarettes smokers also showed higher awareness compared with non-smokers (AOR = 1.73, 95% CI: 1.16–2.57; p = 0.007). Awareness increased with higher educational attainment, with participants educated beyond the compulsory level being more likely to be aware of OSCC (AOR = 3.33, 95% CI: 2.15–5.15; p < 0.001). After adjustment, age and regular dental attendance were not significant predictors.

**Table 4:** Predictors of awareness of oral cancer.

Variable		Awareness of oral cancer, n (%)			Multivariable logistic model (Hosmer and Lemeshow χ^2^ = 9.980, df = 8, p = 0.266)		
		**No**	**Yes**	**P-value**	**AOR**	**95% IC**	**P-value**
**Gender**				< 0.001			
	Male	125 (36.3)	219 (63.7)		Ref.		
	Female	146 (25.6)	424 (74.4)		1.67	1.23-2.26	0.001
**Age group**				0.032			
	< 60 years	144 (26.9)	391 (73.1)		Ref.		
	≥ 60 years	127 (30.7)	252 (66.5)		0.93	0.67-1.29	0.662
**Education**				< 0.001			
	Low	83 (42.6)	112 (57.4)		Ref.		
	Intermediate	127 (32.9)	259 (67.1)		1.54	1.06-2.24	0.023
	High	61 (18.3)	272 (81.7)		3.33	2.15-5.15	< 0.001
**Smoking**				0.028			
	No	204 (31.2)	450 (68.8)		Ref.		
	Cigarettes	42 (22.1)	148 (77.9)		1.73	1.16–2.57	0.007
	E-cigarettes	25 (35.7)	45 (64.3)		0.71	0.41–1.22	0.215
**Dental attendance**				0.020			
	Irregular	113 (34.3)	216 (65.7)		Ref.		
	Regular	158 (27.0)	427 (73.0)		1.14	0.84–1.56	0.403

AOR: adjusted odds ratio; 95% IC: 95% interval confidence.

Table 5 shows variables associated with knowledge of chronic trauma and alcohol consumption as risk factors for OSCC. In the bivariate analysis, knowledge of chronic trauma was associated with age (p = 0.033) and educational level (p < 0.001), but after adjustment only educational level remained statistically significant, with higher educational attainment linked to greater awareness.

**Table 5:** Predictors of knowledge of chronic trauma and alcohol consumption as risk factors for oral cancer.

Chronic trauma as a risk factor for oral cancer							
**Variable**					**Multivariable logistic model** **(Hosmer and Lemeshow χ**^**2**^ **= 6.105, df = 8, p = 0.635)**		
		**No**	**Yes**	**P-value**	**AOR**	**95% IC**	**P-value**
**Gender**				0.311			
	Male	170 (49.4)	174 (50.6)		Ref.		
	Female	262 (46.0)	308 (54.0)		1.07	0.81–1.41	0.652
**Age group**				0.033			
	< 60 years	237 (44.3)	298 (55.7)		Ref.		
	≥ 60 years	195 (51.5)	184 (48.5)		0.93	0.69–1.25	0.638
**Education**				< 0.001			
	Low	121 (62.1)	74 (37.9)		Ref.		
	Intermediate	197 (51.0)	189 (49.0)		1.53	1.06–2.20	0.022
	High	114 (34.2)	219 (65.8)		3.09	2.07–4.61	< 0.001
**Smoking**				0.067			
	No	313 (47.9)	341 (52.1)		Ref.		
	Cigarettes	95 (50.0)	95 (50.0)		0.91	0.65–1.27	0.582
	E-cigarettes	24 (34.3)	46 (65.7)		1.64	0.96–2.79	0.069
**Dental attendance**				0.062			
	Irregular	169 (51.4)	160 (48.6)		Ref.		
	Regular	263 (45.0)	322 (55.0)		1.09	0.82–1.46	0.542
**Alcohol as a risk factor for oral cancer**							
**Variable**					**Multivariable logistic model** **(Hosmer and Lemeshow χ**^**2**^ **= 6.213, df = 8, p = 0.623)**		
		**No**	**Yes**	**P-value**	**AOR**	**95% IC**	**P-value**
**Gender**				0.008			
	Male	143 (41.6)	201 (58.4)		Ref.		
	Female	187 (32.8)	383 (67.2)		1.44	1.09–1.91	0.011
**Age group**				0.119			
	< 60 years	182 (34.0)	353 (66.0)		Ref.		
	≥ 60 years	148 (39.1)	231 (60.9)		0.83	0.62–1.12	0.222
**Education**				0.059			
	Low	73 (37.4)	122 (62.6)		Ref.		
	Intermediate	153 (39.6)	233 (60.4)		0.90	0.62–1.30	0.563
	High	104 (31.2)	229 (68.8)		1.27	0.85–1.90	0.251
**Smoking**				0.256			
	No	226 (34.6)	428 (65.4)		Ref.		
	Cigarettes	78 (41.1)	112 (58.9)		0.76	0.54–1.06	0.104
	E-cigarettes	26 (37.1)	44 (62.9)		0.82	0.49–1.39	0.466
**Dental attendance**							
	Irregular	114 (34.7)	215 (65.3)	0.492	Ref.		
	Regular	216 (36.9)	369 (63.1)		0.79	0.59–1.06	0.114

AOR: adjusted odds ratio; 95% IC: 95% interval confidence

Knowledge of alcohol consumption as a risk factor was associated only with gender, with female participants more likely than males to identify it correctly (AOR = 1.44, 95% CI: 1.09–1.91; p = 0.011).

As detailed in S2 Table, knowledge of smoking as a risk factor for OSCC was consistently high across all subgroups and was not influenced by any of the examined variables. In contrast, knowledge of sunlight exposure as a potential risk factor remained low and showed no association with any participant sociodemographic characteristics or behaviours.

### Need and Sources of additional information

As shown in Table 6, most participants (92.2%) expressed a need for additional information about OSCC. The most frequently preferred sources were pharmacies (67.3%), social media platforms (54.2%), and schools (53.9%), whereas television was selected by only a small minority of respondents (8.8%). Social media was more commonly preferred by younger participants (p < 0.001), while individuals aged over 40 years more frequently favoured schools and television as information sources (p = 0.034 and p < 0.001, respectively). Female participants demonstrated a significantly stronger preference for pharmacies (p = 0.035), and participants with higher educational attainment more often preferred schools and pharmacies (both p = 0.001).

**Table 6:** Frequency distribution of participants’ preferred sources of additional information.

Variable	Category	Percentage (%)
**Need for additional information**	Yes	92.2
	No	7.8
**Preferred information sources**	Pharmacies	67.3
	Social media	54.2
	Schools	53.9
	Newspapers	31.6
	Meeting	25.1
	Television	8.8

## Discussion

This survey provides an overview of current awareness and knowledge of OSCC among adults in North-Western Italy and identifies key sociodemographic determinants of awareness that may inform targeted educational interventions. Although the study population consisted of pharmacy customers rather than a randomly selected sample of the general population, community pharmacies represent a highly frequented healthcare setting in Italy due to their wide territorial coverage and accessibility, thereby making the findings particularly relevant for public health planning [17–22]. This observation is supported by a recent analysis which has shown that in Italy more than 70% of the population visit a pharmacy at least once a month [27]. The study population was heterogeneous and well distributed in terms of age and educational attainment, offering a comprehensive picture of the population-level knowledge on the topic. The predominance of female respondents is consistent with previous studies [28–30] and may reflect women’s greater willingness to participate in survey-based research [31]. Women are also known to use pharmacy and primary healthcare services more frequently than men [17–21].

Overall, approximately 70% of participants reported awareness of OSCC, a figure comparable to that observed in other European studies [28,32] and in recent Italian surveys conducted in Eastern Italy or in the general population [12,30]. This finding indicates a moderate, but still inadequate level of public awareness. Although most participants correctly identified OSCC as a malignant tumour (88.5%), substantial gaps remain in specific knowledge domains, particularly regarding disease aggressiveness, less commonly recognized cancer risk factors and symptoms, and appropriate healthcare pathways. These deficiencies are clinically relevant, as delayed diagnosis and continued exposure to high-risk behaviours contribute to poor OSCC outcomes [4,11]. The observed marked underestimation of oral cancer aggressiveness further supports the notion that awareness of the disease’s existence does not necessarily translate into an adequate understanding of its severity and clinical implications.

In line with earlier studies, awareness of OSCC was significantly greater among women and individuals with higher educational attainment [12,30,33]. This gender disparity may partly reflect women’s generally higher health literacy, greater engagement with preventive healthcare services, and increased attention to oral health-related issues [31,34,35], despite OSCC having been more prevalent among men [1,3]. These findings further reinforce the need for targeted health educational interventions specifically aimed at male populations.

Educational level emerged as a strong independent predictor of OSCC awareness and knowledge of risk factors, including chronic oral trauma. This finding aligns with existing literature indicating that lower educational attainment is associated with reduced cancer awareness and delayed healthcare-seeking behaviour [12,30,33,36]. Individuals with limited education may also have less access to reliable health information and may be more susceptible to misinformation [37]. Notably, among sociodemographic variables, age did not retain a statistically significant association with OSCC awareness in the adjusted model, although bivariate analysis suggested lower awareness among older participants. This may reflect a confounding effect of age in relation to educational level and lifestyle habits, as well as differences in exposure to information sources, with older individuals being more likely to rely on informal channels such as friends and family.

Regarding specific risk factors, tobacco smoking was correctly identified by 97.9% of the participants as the primary cause of OSCC [3,6,38]. Interestingly, current smokers demonstrated higher cancer awareness than non-smokers, a finding also reported in previous studies [30,39,40], possibly due to increased exposure to public health campaigns or healthcare counselling. However, awareness alone does not guarantee behavioural change, and smoking cessation remains a major challenge. Indeed, an Italian survey reported that most smokers continued to smoke despite being aware of its role in OSCC onset [41].

In contrast, awareness of alcohol consumption as risk factor was lower and associated only with gender, with women more likely to recognize its role. Consistent with the present findings, Buykx et al. [42] and Thompsen et al. [43] found that females demonstrated greater awareness of alcohol-related risks for head and neck cancers compared with males. Previous evidence further suggests that alcohol abuse is frequently underestimated as an independent carcinogenic factor, particularly when not combined with tobacco consumption [44].

Knowledge of other established risk factors, such as chronic oral trauma and poor oral hygiene was moderate, while recognition of sunlight exposure as a risk factor was consistently low (18.6%), and in line with percentages reported in previous studies [12,30,45]. These findings highlight critical knowledge gaps, even among individuals with higher education, emphasizing the need for targeted educational initiatives addressing less widely recognized yet clinically relevant behavioural risk factors in oral carcinogenesis.

Regarding clinical presentation, most participants correctly identified non-healing ulcers (82.5%), oral nodules (76.5%), red or white patches (63.6%) as warning signs of OSCC. These findings indicate higher levels of symptom recognition than those previously reported in the Italian population, in which only 4.5% of participants identified both white and red plaques as indicative of OSCC [30]. This observation is clinically relevant, as recognition of early signs is essential for timely consultation and diagnosis [4,6,11]. Nonetheless, approximately one-third of respondents were unaware of the aggressive nature of OSCC, a misconception that may contribute to delayed presentation and poorer prognosis. Moreover, uncertainty about less specific symptoms may further delay diagnosis, particularly when early lesions are asymptomatic or minimally bothersome [4].

When considering healthcare-seeking behaviour, general practitioners, otolaryngologists, and dentists were the most frequently identified professionals to consult in case of suspected OSCC. It is noteworthy that dentists, who play a pivotal role in early screening through routine oral examinations, were not the most selected option. Consistent with the findings by Nocini at al. [30], this suggests limited public awareness of dentists’ role in OSCC diagnosis and highlights the need for targeted educational campaigns aimed at both patients and dentists. Villa et al. reported that fewer than 15% of survey participants had received counselling on oral cancer from their dentists [41].

A relevant finding is the high demand for additional information about OSCC, expressed by most participants. Pharmacies emerged as the most preferred source of information (67.3%), followed by social media (54.2%) and schools (53.9%), with preferences varying according to age, gender, and educational level. These findings suggest that community pharmacies may represent a potentially valuable setting for health education and cancer prevention initiatives related to OSCC. In Italy, more than 20,000 community pharmacies are distributed throughout the territory, often serving as the first point of contact with the National Health Service. Their extended opening hours, accessibility, and established relationships of trust with customers may position pharmacists as potentially valuable contributors to the delivery of preventive messages, the distribution of educational materials, and the promotion of early referral [17–21]. Moreover, current Italian legislation explicitly supports the involvement of community pharmacies in primary, secondary, and tertiary prevention activities [45].

### Strengths and limitations

Although this study benefits from a reasonably large sample size, face-to-face interviews, and regional coverage, several limitations should be acknowledged. The cross-sectional design precludes causal inference, and the use of non-random sampling limits the representativeness and generalizability of the findings. Individuals recruited in community pharmacies may be more engaged with healthcare services, with higher baseline health literacy and exposure to cancer information, while women may be over-represented.

In addition, self-reported data are susceptible to recall and social desirability biases. Recall bias may limit participants’ ability to accurately identify or report less salient OSCC risk factors, whereas social desirability bias, particularly in face-to-face interviews, may lead to over-reporting of awareness of established risk factors such as smoking and alcohol consumption, reflecting perceived expectations rather than true knowledge. Furthermore, although clustering was considered during sample size calculation, pharmacy identifiers were not retained in the anonymized dataset, which precluded cluster-adjusted analyses. This may have led to underestimated standard errors and reduced precision of the reported estimates. In addition, the absence of clustering adjustment may have influenced the robustness of the observed associations, and should be considered when interpreting the findings. Another limitation relates to the questionnaire. Although it was developed based on previously validated instruments and underwent pilot testing and content validation, a comprehensive psychometric validation was not performed.

Taken together, these factors may have resulted in an overestimation of overall awareness and an underestimation of knowledge gaps within the population. In addition, the exploratory nature of the study should be considered when interpreting the observed associations.

### Future directions and implications

Future research should evaluate the effectiveness of structured educational interventions and assess their impact on knowledge retention, healthcare-seeking behaviour, and early diagnosis rates. To maximize effectiveness, such initiatives should be coordinated at a national or regional level, ensuring consistency, scientific accuracy, and integration with dental and medical services. Training pharmacists to recognize early signs of OSCC and to provide evidence-based counselling could further strengthen their contribution to early detection pathways.

## Conclusions

Despite its limitations, this study identifies important gaps in awareness of OSCC among Italian adults, particularly among older individuals and those with lower educational attainment. These findings highlight the need for targeted public health interventions aimed at improving knowledge of OSCC risk factors and early signs.

Community pharmacies may represent a feasible and accessible setting for delivering such interventions. Structured awareness initiatives implemented in these settings could help reach population groups at higher risk and with more frequent contact with healthcare services.

From a public health perspective, integrating OSCC awareness activities into existing prevention strategies may support earlier recognition of symptoms and encourage timely healthcare seeking. Further research is warranted to evaluate the effectiveness of pharmacy-based interventions in improving awareness and related outcomes.

## Supporting information

S1 File Questionnaire. (PDF)

S1 Table Variables associated with knowledge of smoking, sun exposure and oral hygiene as risk factors of oral cancer. (PDF)

S2 File The survey dataset for participants’ responses. (XLSX)
