# Supplementary material for: Knowledge and awareness of oral cancer among adults in North-Western Italy: A cross-sectional questionnaire-based survey in community pharmacies
Source: PLoS One. 2026 Jul 24;21(7):e0354509. doi: 10.1371/journal.pone.0354509 (PMC13399452; doi:10.1371/journal.pone.0354509)
Supplement: S1 File — (PDF) [file pone.0354509.s001.pdf]

# Demographic Information

**Age**
→ *Your answer*

**Sex**

- Male
- Female

# General Information

**What is your highest level of education?**

- Middle school
- High school
- Bachelor’s/Master’s degree

**Do you smoke?**

- Yes
- No
- Electronic cigarettes or similar (IQOS, e-cigarettes)

**Do you attend dental check-ups?**

- Only when needed
- Every 6 months
- Once a year

# Awareness about Oral Cancer

**Have you ever heard of oral cancer?**

- Yes
- No

**If yes, where did you hear about it?** *(Multiple choice)*

- Television
- Internet
- Doctor
- Pharmacist
- Friends or relatives
- Other: ______

# Risk Factors

**Which of the following are considered risk factors for oral cancer?** *(Multiple choice)*

- Smoking
- Alcohol consumption
- Poor oral hygiene
- Sun exposure
- Repeated trauma to the inner surface of the mouth

# Symptoms

**Which of the following are considered symptoms of oral cancer?** *(Multiple choice)*

- Unexplained loosening of teeth
- Darkening of teeth
- Changes in speech
- Difficulty moving the jaw
- Pain or difficulty when swallowing
- Dizziness
- Persistent numbness of the lips or tongue
- Insomnia
- White or red patches in the mouth or on the tongue
- Persistent lumps in the mouth
- Persistent lumps in the neck glands
- Hair loss
- Unexplained weight loss
- Persistent cold
- Painful mouth ulcers that do not heal within a few weeks

# Knowledge

**Which of the following tumors are considered malignant?** *(Multiple choice)*

- Uterine fibroma
- Liver carcinoma
- Lung cancer
- Lipoma
- Angioma
- Oral cancer
- None of the above

**Do you know that oral cancer can be fatal?**

- Yes, it is a very aggressive cancer
- No, it is a less aggressive cancer
- I don’t know

**At what age does oral cancer typically occur?**

- 20–40 years
- 40–60 years
- 60–90 years
- Over 90 years

# Healthcare Behavior

**Whom would you consult if you suspected oral cancer or wished to undergo a preventive check-up?** *(Multiple choice)*

- Pharmacist
- General practitioner
- Dentist
- Dermatologist
- ENT specialist (Otolaryngologist)
- Oncologist

# Information Dissemination

**Which communication channels do you think are most effective for disseminating information about oral cancer?** *(Multiple choice)*

- Public meetings
- Pharmacies
- Internet and social media
- Newspapers
- Schools
- There is no need for further information
- Other: ______
