# Supplementary material for: Knowledge and awareness of oral cancer among adults in North-Western Italy: A cross-sectional questionnaire-based survey in community pharmacies
Source: PLoS One. 2026 Jul 24;21(7):e0354509. doi: 10.1371/journal.pone.0354509 (PMC13399452; doi:10.1371/journal.pone.0354509)
Supplement: S1 Table — (PDF) [file pone.0354509.s002.pdf]

S2 Table. Variables associated with knowledge of smoking habits, sunlight exposure and oral hygiene as risk factors of oral cancer.

Smoking as a risk factor for oral cancer							
**Variable**					**Multivariable logistic model (Hosmer and Lemeshow χ^2 = 8.815, df = 8, p = 0.266)**		
		**No**	**Yes**	**P-value**	**AOR**	**95% IC**	**P-value**
**Gender**				0.065			
	Male	11 (3.2)	333 (96.8)		Ref.		
	Female	8 (1.4)	562 (98.6)		2.37	0.92–6.07	0.073
**Age group**				0.598			
	< 60 years	10 (1.9)	525 (98.1)		Ref.		
	≥ 60 years	9 (2.4)	370 (97.6)		0.91	0.33–2.49	0.847
**Education**				0.858			
	Low	4 (2.1)	191 (97.9)		Ref.		
	Intermediate	7 (1.8)	379 (98.2)		1.00	0.28–3.59	0.996
	High	8 (2.4)	325 (97.6)		0.65	0.17–2.47	0.527
**Smoking**							
	No	17 (2.6)	637 (97.4)	0.195	Ref.		
	Cigarettes	1 (0.5)	189 (99.5)		5.59	0.73–22.7	0.197
	E-cigarettes	1 (1.4)	69 (98.6)		1.81	0.23–14.2	0.574
**Dental attendance**				0.297			
	Irregular	9 (2.7)	320 (97.3)		Ref.		
	Regular	10 (2.7)	575 (98.3)		1.56	0.61–4.04	0.357
**Sunlight exposure as a risk factor for oral cancer**							
**Variable**					**Multivariable logistic model (Hosmer and Lemeshow χ^2 = 5.485, df = 8, p = 0.705)**		
		**No**	**Yes**	**P-value**	**AOR**	**95% IC**	**P-value**
**Gender**				0.382			
	Male	285 (82.8)	59 (17.2)		Ref.		
	Female	459 (80.5)	111 (19.5)		1.16	0.81–1.66	0.411
**Age group**				0.795			
	< 60 years	437 (81.7)	98 (18.3)		Ref.		
	≥ 60 years	307 (81.0)	72 (19.0)		0.96	0.66–1.39	0.846
**Education**				0.613			
	Low	155 (79.5)	40 (20.5)		Ref.		
	Intermediate	313 (81.1)	73 (18.9)		0.90	0.58–1.41	0.650
	High	276 (82.9)	57 (17.1)		0.80	0.49–1.31	0.374
**Smoking**				0.139			
	No	525 (80.7)	129 (19.7)		Ref.		
	Cigarettes	164 (86.3)	26 (13.7)		0.65	0.41–1.03	0.067
	E-cigarettes	55 (78.6)	15 (21.4)		1.11	0.60–2.06	0.730
**Dental attendance**				0.500			
	Irregular	264 (80.2)	65 (19.8)		Ref.		
	Regular	480 (82.1)	105 (17.9)		0.88	0.61–1.26	0.483

Poor oral hygiene as a risk factor for oral cancer							
**Variable**					**Multivariable logistic model (Hosmer and Lemeshow χ^2 = 7.262, df = 8, p = 0.509)**		
		**No**	**Yes**	**P-value**	**AOR**	**95% IC**	**P-value**
**Gender**				0.611			
	Male	160 (46.5)	184 (53.5)		Ref.		
	Female	275 (48.2)	295 (51.8)		0.92	0.70–1.21	0.557
**Age group**				0.153			
	< 60 years	244 (45.6)	291 (54.4)		Ref.		
	≥ 60 years	191 (50.4)	188 (49.6)		0.80	0.60–1.07	0.130
**Education**							
	Low	88 (45.1)	107 (54.9)	0.204	Ref.		
	Intermediate	197 (51.0)	189 (49.0)		0.75	0.52–1.07	0.109
	High	150 (45.0)	183 (55.0)		0.93	0.63–1.36	0.695
**Smoking**				0.705			
	No	313 (47.9)	341 (52.1)		Ref.		
	Cigarettes	92 (48.4)	98 (51.6)		0.94	0.68–1.31	0.722
	E-cigarettes	30 (42.9)	40 (57.1)		1.19	0.72–1.97	0.503
**Dental attendance**				0.441			
	Irregular	151 (45.9)	178 (54.1)		Ref.		
	Regular	284 (48.5)	301 (51.5)		0.88	0.67–1.67	0.378

AOR: adjusted odds ratio; 95% IC: 95% interval confidence.
