# Supplementary material for: Knowledge and awareness of oral cancer among adults in North-Western Italy: A cross-sectional questionnaire-based survey in community pharmacies
Source: PLoS One. 2026 Jul 24;21(7):e0354509. doi: 10.1371/journal.pone.0354509 (PMC13399452; doi:10.1371/journal.pone.0354509)
Supplement: Attachment — Submitted filename: Response Letter.pdf [file pone.0354509.s005.pdf]

Authors' Response to the Reviewers Comments

Journal: *PLOS ONE*

Title of Paper: Knowledge and awareness of oral cancer among adults in North-Western Italy: a cross-sectional questionnaire-based survey in community pharmacies.

Authors: Francesca Baratta, Federica Romano, Paola Brusa, Paolo Giacomo Arduino

EDITOR:

Thank you for submitting your manuscript to PLOS ONE. After careful consideration, we feel that it has merit but does not fully meet PLOS ONE's publication criteria as it currently stands. Therefore, we invite you to submit a revised version of the manuscript that addresses the points raised during the review process.

We sincerely thank the Editor and reviewers for their insightful comments and constructive feedback, which have significantly improved the clarity and overall quality of our manuscript. We have revised the manuscript to ensure full compliance with *PLOS ONE* style requirements, including file naming conventions, and we have provided an English-language version of the dataset as well as a translated version of the questionnaire as Supporting Information. We have also carefully reviewed all publications suggested by the reviewers and incorporated relevant citations into the revised manuscript where appropriate.

REVIEWER: 1

COMMENT 1:

While community pharmacies provide a practical recruitment setting, they may not be representative of the general population. The discussion should be expanded to address how this sampling approach may influence health literacy levels, gender distribution, and healthcare-seeking behaviour.

Response:

Thank you for your valuable comment. We agree that recruitment via community pharmacies may limit generalizability. Pharmacy attendees are more likely to be engaged with healthcare services and may therefore have higher baseline health literacy and greater exposure to cancer awareness information than the general population. This could lead to an overestimation of cancer awareness levels and more proactive help-seeking behaviors in our sample. In addition, women may be over-represented due to their higher use of pharmacy and primary care services. These factors have been acknowledged in the Discussion, and we have clarified that the findings should be interpreted with caution when generalizing to less healthcare-engaged populations.

COMMENT 2:

The manuscript emphasizes pharmacies as key intervention sites; however, this conclusion was not directly tested. Statements should be reframed to reflect that pharmacies represent a potential, rather than a proven, channel for interventions. Consider using phrasing such as: "These findings suggest potential utility..." instead of making definitive claims.

Response:

We thank the reviewer for this helpful suggestion. We have revised the manuscript to avoid definitive statements regarding the role of community pharmacies as intervention sites and to frame pharmacies more cautiously as a potentially valuable setting for future awareness and prevention initiatives.

COMMENT 3:

Although the manuscript mentions piloting of the questionnaire, it lacks sufficient detail. Additional information should be provided regarding validation procedures (e.g., reliability testing), including metrics such as Cronbach's alpha, if available. It should also be elucidated whether the questionnaire was derived from previously validated instruments or developed specifically for this study.

Response:

We thank the reviewer for the suggestion. More details have been added to the text to clarify how the questionnaire was developed starting from previously validated instruments. It was also explained how it was reviewed by expert reviewers and tested before being used in the study. Since the questionnaire mainly included factual and dichotomous items assessing awareness and knowledge rather than psychometric constructs, internal consistency measures such as Cronbach's alpha were not considered appropriate.

COMMENT 4:

The use of self-reported data introduces potential recall and social desirability biases. The discussion should be expanded and further elaborated to ensure that readers understand exactly how these biases may have influenced responses, particularly regarding smoking awareness and knowledge of risk factors.

Response:

We thank the reviewer for this important comment. We agree that self-reported data may introduce recall and social desirability biases, which warrant further clarification. We have expanded the Discussion to explicitly describe how these biases may have influenced participants’ responses, particularly in relation to knowledge of oral cancer risk factors. The revised text also clarifies the potential direction of these biases, including possible overestimation of awareness and socially desirable reporting of health-related knowledge and behaviors.

COMMENT 5:

The authors should explain whether non-response rates were recorded and, if so, how they were handled in the analysis.

Response:

We thank the reviewer for this important comment. Non-response was recorded during fieldwork within each pharmacy. Eligible customers who declined participation or were unavailable were not included in the study, and recruitment proceeded consecutively until the predefined cluster targets were reached. Non-response was minimal (overall response rate: 95.1%), and the achieved sample size exceeded the minimum required sample size. Although individual-level information was not systematically collected for those who declined participation, the overall response rate was high (95.1%), which may have reduced the likelihood of substantial non-response bias. Therefore, no statistical adjustment for non-response was applied. All analyses were conducted using complete-case data from enrolled participants. This information has been added to the revised manuscript to improve transparency regarding data collection and handling procedures.

COMMENT 6:

Table captions should be more detailed to ensure they are fully self-explanatory. All abbreviations (e.g., OR, CI) should be defined within the captions. Additionally, large tables (particularly Table 4) could be simplified to improve readability.

Response:

Thank you for this valuable suggestion. We have revised all table captions to make them more detailed and self-explanatory. Specifically, all abbreviations used in the tables, including OR (Odds Ratio) and CI (Confidence Interval), are now fully defined within the respective captions. In addition, we reviewed the formatting and structure of the larger tables, particularly Table 4, and simplified their presentation to improve readability and clarity.

COMMENT 7:

Minor grammatical and stylistic issues are present throughout the manuscript. Consistent terminology should be ensured (e.g., OSCC vs. oral cancer), and typographical inconsistencies (e.g., "OCSS") should be corrected. Some sentences in the Discussion are overly complex and could be streamlined for straightforwardness. Concise can be improved by reducing minor redundancies.

Response: We thank the reviewer for this helpful observation. We have carefully revised the manuscript to address grammatical and stylistic issues throughout the text. Consistent terminology has now been ensured, and typographical errors have been corrected. In addition, the Discussion section has been edited to improve readability by simplifying overly complex sentences and removing minor redundancies, thereby enhancing overall clarity and conciseness.

COMMENT 8:

There is limited contextualization of the findings within the existing literature. The authors should strengthen their engagement with prior studies by comparing results and methodologies. Incorporating a conceptual framework would further strengthen the theoretical underpinning of the study.

Response:

We thank the reviewer for this important comment. In the revised manuscript, we have strengthened the contextualization of our findings by comparing them with previous national and international studies investigating oral cancer awareness and related sociodemographic determinants. We have also expanded the discussion on health literacy, preventive behaviour, and healthcare engagement as factors potentially influencing awareness and healthcare-seeking attitudes related to oral cancer.

COMMENT 9:

The conclusion should be revised to provide a more assertive interpretation of the findings while clearly emphasizing implications for public health policy and intervention strategies.

Response:

We thank the reviewer for his/her valuable comment. In response, we have revised the Conclusion section to provide a more assertive interpretation of the present findings and to more clearly highlight their implications for public health policy and intervention strategies.

COMMENT 10:

The authors are also encouraged to provide the survey instrument and consider including supplementary materials detailing data handling procedures and variable coding to enhance reproducibility. Additional information on statistical model diagnostics would also strengthen the methodological transparency.

Response:

Thank you for this valuable suggestion. In response, we have provided the English versions of both the dataset and the questionnaire to improve transparency and reproducibility. We have also incorporated additional information on statistical model diagnostics in the revised manuscript to strengthen methodological clarity.

REVIEWER: 2

COMMENT 1:

Minor comments:
Correct OCSS in lines 273, 293, 332, 336, and 364 into OSCC. Table 1 correct 22,2 into 22.2. There is an updated version as of 2021 for reference 2.

Response: Thank you for the comments. We have corrected "OCSS" to "OSCC" in lines 273, 293, 332, 336, and 364, and "22,2" to "22.2" in Table 1. Reference 2 has been updated to the 2021 version.

COMMENT 2:

Questionnaire development and validation
The authors state in the manuscript that the questionnaire was piloted on a small group; however, no details are provided regarding its development or validation. No validated instrument is referenced, no psychometric evaluation is reported, and no description of item generation or expert involvement is included. This represents a critical methodological issue because the questionnaire constitutes the core of the study.

Response:

We thank the reviewer for the suggestion. More details have been added to the text to clarify how the questionnaire was developed starting from previously validated instruments. It was also explained how it was reviewed by expert reviewers and tested before being used in the study. Since the questionnaire mainly included factual and dichotomous items assessing awareness and knowledge rather than psychometric constructs, internal consistency measures such as Cronbach's alpha were not considered appropriate.

COMMENT 3:

Sampling strategy and representativeness
Participants were recruited from community pharmacies, but the sampling method is not described. The selection of pharmacies is unclear, the recruitment process is not defined, and the claim of regional representativeness is not justified. This raises concerns regarding potential selection bias and limits the generalizability of the findings.

Response:

We thank the reviewer for this helpful observation. The manuscript has been carefully revised to detail how pharmacy recruitment was conducted to ensure the variability across provinces and urban-rural settings.

COMMENT 4:

Response rate and recruitment flow
No information is provided regarding the number of individuals approached, the refusal rate, or the response rate. The absence of these details limits the assessment of potential non-response bias and transparency of the recruitment process.

Response:

Thank you for the comment. We have revised the Methods and Results sections to provide additional details on the recruitment flow. Eligible pharmacy customers were consecutively approached, and 914 out of 961 agreed to participate, yielding an overall response rate of 95.1%. Although individual-level information was not systematically collected for those who declined participation, the high response rate may have reduced the likelihood of substantial non-response bias. This limitation has been acknowledged in the revised manuscript.

COMMENT 5:

Survey administration and bias
The study used interviewer-administered questionnaires, which introduces a risk of social desirability bias. However, no details are provided regarding standardization procedures or interviewer calibration. This lack of information makes it difficult to assess the reliability of data collection.

Response:

Thank you for this important comment. We would like to clarify that all interviewers underwent prior calibration and were trained using standardized procedures before data collection. This included guidance on consistent question delivery and minimizing interviewer influence. We will revise the manuscript to explicitly describe these steps to improve transparency and allow better assessment of the reliability of data collection.

COMMENT 6:

Statistical analysis
Several concerns exist regarding the statistical approach. Variable selection based solely on univariate significance is not methodologically robust and may lead to biased models. In addition, model diagnostics are not reported in sufficient detail. Although clustering was considered in the sample size calculation, the dataset does not appear to include any clustering variable (like pharmacy identifier), and no adjustment for clustering is reported in the analysis. Table 4 presents inconsistent reporting of regression results across outcomes. While odds ratios, confidence intervals, and p-values are fully reported for awareness of oral cancer, they are only partially reported for some outcomes (alcohol consumption and chronic trauma/irritation as risk factors) and not reported at all for others (smoking, sunlight exposure, and poor oral hygiene as risk factors). This inconsistency makes it unclear whether multivariable logistic regression analyses were performed for all outcomes, as stated in the methods, or only for selected variables.

Response:

Thank you for these important comments. We revised the manuscript to improve the clarity and robustness of the statistical analysis. Variable selection for multivariable logistic regression is now described as being based on both theoretical relevance and prior literature, rather than solely on univariate significance. Additional information on goodness-of-fit assessment has also been added.
We further clarified that clustering was considered during sample size calculation; however, pharmacy identifiers were not retained in the anonymized dataset, preventing clustering adjustment in the final analyses. This limitation is now acknowledged in the manuscript. However, given the relatively large number of participating pharmacies and the exploratory nature of the study, the impact on the estimated associations is expected to be limited. Finally, Table 4 has been revised to ensure consistent reporting across all outcomes. Odds ratios (ORs), 95% confidence intervals (CIs), and p-values are now uniformly presented for all multivariable logistic regression analyses, and the

Methods and Results sections clearly state that these analyses were performed for all specified outcomes.

COMMENT 7:

Outcome definition
The definition of "awareness" is unclear, as it is not specified whether it is treated as a binary variable or derived from a composite score, nor are any cut-off criteria described. This lack of clarity affects the interpretability of the results.

Response:

Thank you for highlighting this point. We agree that the definition of "awareness" required clarification. In our study, awareness was assessed using a single item from the questionnaire and treated as a binary variable. Specifically, participants were classified as "aware" if they responded "yes" to the question whether they had previously heard of oral cancer, and "not aware" if they responded "no." No composite score or cut-off criteria were applied. We have now clarified this definition in the Methods section to improve transparency and interpretability.

COMMENT 8:

Missing data handling
No description of missing data handling is provided, which limits transparency and reproducibility. It is clear that missing data were present (see S1 file, under column H) and, if so, how it was managed.

Response:

Thank you for your observation. We would like to clarify that there were no missing data in our dataset. The apparent incompleteness in column H (see S1 File) is due to the structure of the questionnaire rather than missing responses. Specifically, only participants who indicated awareness of cancer were subsequently asked to identify their source of information. Therefore, entries in column H are blank for participants who did not report cancer awareness, as this question was not applicable to them.

COMMENT 9:

Limitation section
The limitations section is incomplete and does not fully reflect the methodological constraints of the study. Important issues such as non-random sampling, use of a non-validated questionnaire, potential interviewer and social desirability bias, and lack of adjustment for clustering are not adequately discussed.

Response:

Thank you for this important comment. We agree that the limitations section should more comprehensively reflect the methodological constraints of the study. In response, we have revised the manuscript to explicitly acknowledge the use of non-random sampling, which may limit the generalizability of our findings. We have also clarified the potential for social desirability bias, given the nature of data collection, and how these factors may have influenced participants' responses. We also clarified that, although the questionnaire underwent pilot testing and expert content validation

procedures, it was specifically adapted for this study and did not undergo full psychometric validation. Finally, we have included a statement regarding the lack of adjustment for clustering in the analysis, which may have implications for the precision of our estimates.

COMMENT 10:

Self-citation
A high proportion of the references cited in the manuscript appear to be authored by the same researchers in this manuscript (approximately 15 out of 44 references). While self-citation is acceptable when scientifically justified, this proportion is relatively high and may introduce bias or limit the breadth of the literature review.

Response:

Thank you for your observation. The bibliography has been revised to be more appropriate.
