# Supplementary material for: Knowledge and awareness of oral cancer among adults in North-Western Italy: A cross-sectional questionnaire-based survey in community pharmacies
Source: PLoS One. 2026 Jul 24;21(7):e0354509. doi: 10.1371/journal.pone.0354509 (PMC13399452; doi:10.1371/journal.pone.0354509)
Supplement: Attachment — Submitted filename: REVIEWER CONSIDERATIONS.pdf [file pone.0354509.s006.pdf]

REVIEWER CONSIDERATIONS

The authors investigate public knowledge and awareness of Oral Squamous Cell Carcinoma (OSCC) and its associated risk factors among adults in Northwest Italy. Using a cross-sectional survey of 914 participants recruited through community pharmacies, the study demonstrates that although 70.4% of respondents were aware of OSCC, important knowledge gaps remain regarding its aggressiveness and less-recognized risk factors, including alcohol consumption and sun exposure. The findings identify gender, educational level, and smoking status as significant predictors of awareness.

The manuscript is well-grounded in the existing literature. The authors appropriately contextualize their findings by comparing them with previous Italian and European studies. While national-level evidence exists, regional data remain fragmented; therefore, the study provides an insight into a specific geographic area and highlights the potential role of community pharmacies as settings for public health initiatives.

The presentation of results is clear and effective. Tables 1–6 are logically organized and directly support the study findings. In response to previous reviewer comments, table captions have been expanded, and abbreviations such as OR (Odds Ratio) and CI (Confidence Interval) are clearly defined. The use of frequencies and adjusted odds ratios (AORs) is appropriate for the cross-sectional design and analytical objectives.

The methodology is soundly suited to the research question. The face-to-face, observational cross-sectional survey design is appropriate for assessing awareness and knowledge within the target population. The sample size was adequately calculated (target n = 910; achieved n = 914), and potential confounding factors were addressed through multivariable logistic regression analyses. The inclusion of the full dataset (S3 File) and the translated questionnaire (S1 File) enhances transparency and reproducibility.

The authors provide a balanced discussion of the study limitations. They acknowledge the possibility of selection bias arising from pharmacy-based recruitment, as participants may be more health-conscious than the general population. Potential recall and social desirability biases inherent to face-to-face interviews are also appropriately discussed. Furthermore, the authors transparently explain that although clustering was considered during sample size estimation, cluster-adjusted analyses could not be performed because pharmacy identifiers were removed during anonymization.

The study adheres to ethical standards, having received approval from the Bioethics Committee of the University of Turin (Protocol No. 0494208), and written informed consent was obtained from all participants. Reporting follows STROBE guidelines for observational studies.

Importantly, the conclusions are supported by the results. The authors have revised earlier overstatements and now appropriately describe community pharmacies as a

potential, rather than proven, setting for future interventions. The finding that 92.2% of respondents expressed a desire for additional information and 67.3% identified pharmacies as their preferred information source provides practical guidance for future awareness campaigns and public health strategies.

Overall, the manuscript is well-structured, methodologically rigorous, transparent in its reporting, and responsive to previous reviewers' concerns. It makes a meaningful contribution to the literature on OSCC awareness and offers actionable insights for public health practice.

Recommendation: ACCEPT.
