# Supplementary material for: Knowledge and awareness of oral cancer among adults in North-Western Italy: A cross-sectional questionnaire-based survey in community pharmacies
Source: PLoS One. 2026 Jul 24;21(7):e0354509. doi: 10.1371/journal.pone.0354509 (PMC13399452; doi:10.1371/journal.pone.0354509)
Supplement: Attachment — Submitted filename: REVIEW~1.DOC [file pone.0354509.s004.DOC]

This manuscript presents a cross-sectional survey assessing adults' knowledge of oral squamous cell carcinoma (OSCC) in North-Western Italy. Using a structured, interviewer-administered questionnaire in community pharmacies (n = 914), the authors evaluate awareness levels, knowledge of risk factors and symptoms, and demographic predictors. The study also explores the potential role of community pharmacies as venues for public health education. This is a well-conducted and clearly written study that addresses an important gap in oral cancer research. The manuscript is methodologically sound, with appropriate statistical analysis and a strong discussion.

However, several areas require clarification and refinement to improve rigor and presentation. While community pharmacies provide a practical recruitment setting, they may not be representative of the general population. The discussion should be expanded to address how this sampling approach may influence health literacy levels, gender distribution, and healthcare-seeking behavior. The manuscript emphasizes pharmacies as key intervention sites; however, this conclusion was not directly tested. Statements should be reframed to reflect that pharmacies represent a potential, rather than a proven, channel for interventions. Consider using phrasing such as: “These findings suggest potential utility…” instead of making definitive claims. Although the manuscript mentions piloting of the questionnaire, it lacks sufficient detail. Additional information should be provided regarding validation procedures (e.g., reliability testing), including metrics such as Cronbach’s alpha, if available. It should also be elucidated whether the questionnaire was derived from previously validated instruments or developed specifically for this study. The use of self-reported data introduces potential recall and social desirability biases. The discussion should be expanded and further elaborated to ensure that readers understand exactly how these biases may have influenced responses, particularly regarding smoking awareness and knowledge of risk factors. The authors should explain whether non-response rates were recorded and, if so, how they were handled in the analysis. Table captions should be more detailed to ensure they are fully self-explanatory. All abbreviations (e.g., OR, CI) should be defined within the captions. Additionally, large tables (particularly Table 4) could be simplified to improve readability. Minor grammatical and stylistic issues are present throughout the manuscript. Consistent terminology should be ensured (e.g., OSCC vs. oral cancer), and typographical inconsistencies (e.g., “OCSS”) should be corrected. Some sentences in the Discussion are overly complex and could be streamlined for straightforwardness. With minor revisions to improve clarity, appropriately temper conclusions, and expand methodological detail, the manuscript would be suitable for publication.  The manuscript provides meaningful insights into oral cancer awareness and highlights important public health opportunities. The abstract and introduction are generally clear and informative; however, revisions are recommended to enhance analytical depth, conceptual transparency and overall impact. There is limited contextualization of the findings within the existing literature. The authors should strengthen their engagement with prior studies by comparing results and methodologies. Incorporating a conceptual framework would further strengthen the theoretical underpinning of the study. The conclusion should be revised to provide a more assertive interpretation of the findings while clearly emphasizing implications for public health policy and intervention strategies. Concise can be improved by reducing minor redundancies. The authors are also encouraged to provide the survey instrument and consider including supplementary materials detailing data handling procedures and variable coding to enhance reproducibility. Additional information on statistical model diagnostics would also strengthen the methodological transparency.
