# Supplementary material for: Knowledge and awareness of oral cancer among adults in North-Western Italy: A cross-sectional questionnaire-based survey in community pharmacies
Source: PLoS One. 2026 Jul 24;21(7):e0354509. doi: 10.1371/journal.pone.0354509 (PMC13399452; doi:10.1371/journal.pone.0354509)
Supplement: Attachment — Submitted filename: Response Letter.docx [file pone.0354509.s007.docx]

# Authors’ Response to the Reviewers Comments

***Journal:***	*PLOS ONE*

***Title of Paper:***	Knowledge and awareness of oral cancer among adults in North-Western Italy: a cross-sectional questionnaire-based survey in community pharmacies.

***Authors:***	Francesca Baratta, Federica Romano, Paola Brusa, Paolo Giacomo Arduino

**EDITOR:**

*The manuscript is considered scientifically sound and has improved considerably following revision. The authors are requested to address a few minor issues related to reporting transparency, methodological limitations, and reference selection. These points do not affect the overall conclusions of the study but should be considered to further enhance the clarity and rigor of the manuscript. Please refer to the reviewer’s comments below for specific recommendations.*

We sincerely thank the Editor and reviewers for the positive evaluation of our manuscript and for recognizing the substantial improvements made during the revision process. We have carefully considered all comments and have revised the manuscript accordingly. Specifically, we have strengthened adherence to reporting guidelines, expanded the discussion of methodological limitations, clarified aspects of questionnaire development, and further reviewed the reference list to ensure a balanced and comprehensive representation of the relevant literature.

**REVIEWER: 1**

COMMENT: *The authors investigate public knowledge and awareness of Oral Squamous Cell Carcinoma (OSCC) and its associated risk factors among adults in Northwest Italy. Using a cross-sectional survey of 914 participants recruited through community pharmacies, the study demonstrates that although 70.4% of respondents were aware of OSCC, important knowledge gaps remain regarding its aggressiveness and less-recognized risk factors, including alcohol consumption and sun exposure. The findings identify gender, educational level, and smoking status as significant predictors of awareness.*

*The manuscript is well-grounded in the existing literature. The authors appropriately contextualize their findings by comparing them with previous Italian and European studies. While national-level evidence exists, regional data remain fragmented; therefore, the study provides an insight into a specific geographic area and highlights the potential role of community pharmacies as settings for public health initiatives.*

*The presentation of results is clear and effective. Tables 1–6 are logically organized and directly support the study findings. In response to previous reviewer comments, table captions have been expanded, and abbreviations such as OR (Odds Ratio) and CI (Confidence Interval) are clearly defined. The use of frequencies and adjusted odds ratios (AORs) is appropriate for the cross-sectional design and analytical objectives.*

*The methodology is soundly suited to the research question. The face-to-face, observational cross-sectional survey design is appropriate for assessing awareness and knowledge within the target population. The sample size was adequately calculated (target n = 910; achieved n = 914), and potential confounding factors were addressed through multivariable logistic regression analyses. The inclusion of the full dataset (S3 File) and the translated questionnaire (S1 File) enhances transparency and reproducibility.*

*The authors provide a balanced discussion of the study limitations. They acknowledge the possibility of selection bias arising from pharmacy-based recruitment, as participants may be more health-conscious than the general population. Potential recall and social desirability biases inherent to face-to-face interviews are also appropriately discussed. Furthermore, the authors transparently explain that although clustering was considered during sample size estimation, cluster-adjusted analyses could not be performed because pharmacy identifiers were removed during anonymization. The study adheres to ethical standards, having received approval from the Bioethics Committee of the University of Turin (Protocol No. 0494208), and written informed consent was obtained from all participants. Reporting follows STROBE guidelines for observational studies.*

*Importantly, the conclusions are supported by the results. The authors have revised earlier overstatements and now appropriately describe community pharmacies as a potential, rather than proven, setting for future interventions. The finding that 92.2% of respondents expressed a desire for additional information and 67.3% identified pharmacies as their preferred information source provides practical guidance for future awareness campaigns and public health strategies.*

*Overall, the manuscript is well-structured, methodologically rigorous, transparent in its reporting, and responsive to previous reviewers' concerns. It makes a meaningful contribution to the literature on OSCC awareness and offers actionable insights for public health practice.*

## Response:

We sincerely thank the reviewer for the positive and constructive evaluation of our manuscript. We are pleased that the reviewer considers the study a meaningful contribution to the literature on oral squamous cell carcinoma awareness and recognizes the potential public health implications of our findings. The reviewer's constructive comments and recommendations have strengthened the clarity and quality of the manuscript.

**REVIEWER: 2**

## COMMENT 1:

*Although the revised manuscript references the STROBE guideline and reporting quality has substantially improved, formal adherence to EQUATOR reporting standards remains incomplete. In particular, a completed STROBE checklist was not provided, and alignment with CROSS remains limited.*

*Recommendation:*

*The authors are encouraged to submit a completed STROBE checklist and improve explicit alignment with CROSS recommendations to further strengthen methodological transparency and reporting rigor. PLOS ONE instructions are clear: Methodological integrity and transparency come first.*

## Response: We appreciate this important observation. In response, we have prepared and submitted a completed STROBE checklist as supplementary material, with page references indicating where each reporting item is addressed in the manuscript. We have also reviewed the manuscript against the CROSS recommendations and further strengthened the reporting of study design, sampling procedures, questionnaire administration, and statistical methods. These revisions have enhanced the methodological transparency and reporting rigor in accordance with EQUATOR Network guidance and PLOS ONE reporting requirements.

## COMMENT 2:

*Questionnaire development and validation*

*The manuscript still lacks sufficient information regarding item adaptation from previous instruments and does not include full psychometric validation procedures.*

*Recommendation:*

*The authors should expand the limitation section to include that the questionnaire did not undergo full psychometric validation.*

## Response:

We thank the reviewer for this valuable comment. We have expanded the description of questionnaire development in the Methods section, including clarification of the sources from which questionnaire items were adapted and the process used for content review and refinement. We agree that the questionnaire did not undergo a complete psychometric validation process. Therefore, we have explicitly acknowledged this limitation in the revised manuscript.

## COMMENT 3:

*Clustering and statistical analysis*

*The potential effect of unadjusted clustering on estimate precision remains important.*

*Recommendation:*

*The limitation regarding the lack of clustering adjustment should be stated more cautiously, specifically acknowledging that standard errors and confidence intervals may potentially be underestimated.*

## Response:

We appreciate this observation and agree that this issue warrants clearer discussion. Accordingly, we have revised the Limitations section to explicitly acknowledge that clustering effects were not formally accounted for in the analysis. We now state that the absence of clustering adjustment may have resulted in underestimated standard errors and confidence intervals, potentially affecting the precision of some estimates. This limitation has been added to ensure a more balanced interpretation of the study findings.

## COMMENT 4:

*Self-Citation and number of references:*

*Instead of removing references, replace or increase them. Self-cited references have been reduced, but are still high unless scientifically justified.*

## Response:

We thank the reviewer for this suggestion. We carefully re-evaluated the reference list and incorporated additional relevant references to provide a more comprehensive contextual framework. Self-citations were reviewed individually and retained only when directly relevant to the study objectives, methodology, or interpretation of findings. We believe the revised reference list now achieves a more balanced representation of the available literature while maintaining appropriate citation of prior work that is specifically pertinent to the present study.
