# Supplementary material for: Knowledge and awareness of oral cancer among adults in North-Western Italy: A cross-sectional questionnaire-based survey in community pharmacies
Source: PLoS One. 2026 Jul 24;21(7):e0354509. doi: 10.1371/journal.pone.0354509 (PMC13399452; doi:10.1371/journal.pone.0354509)
Supplement: Peer review history

# Peer review: Decision Letter 0

**DOI:** 10.1371/journal.pone.0354509.r001

**Authors:** Ronell Bologna-Molina

21 Apr 2026

PONE-D-26-13277

Knowledge and awareness of oral cancer among adults in North-Western Italy: a cross-sectional questionnaire-based survey in community pharmacies.

PLOS One

Dear Dr. Romano,

Thank you for submitting your manuscript to PLOS ONE. After careful consideration, we feel that it has merit but does not fully meet PLOS ONE’s publication criteria as it currently stands. Therefore, we invite you to submit a revised version of the manuscript that addresses the points raised during the review process.

Please submit your revised manuscript by Jun 05 2026 11:59PM. If you will need more time than this to complete your revisions, please reply to this message or contact the journal office at plosone@plos.org. When you're ready to submit your revision, log on to https://www.editorialmanager.com/pone/ and select the 'Submissions Needing Revision' folder to locate your manuscript file.

Please include the following items when submitting your revised manuscript:

- A letter that responds to each point raised by the academic editor and reviewer(s). You should upload this letter as a separate file labeled 'Response to Reviewers'.
- A marked-up copy of your manuscript that highlights changes made to the original version. You should upload this as a separate file labeled 'Revised Manuscript with Track Changes'.
- An unmarked version of your revised paper without tracked changes. You should upload this as a separate file labeled 'Manuscript'.

If you would like to make changes to your financial disclosure, please include your updated statement in your cover letter. Guidelines for resubmitting your figure files are available below the reviewer comments at the end of this letter.

If applicable, we recommend that you deposit your laboratory protocols in protocols.io to enhance the reproducibility of your results. Protocols.io assigns your protocol its own identifier (DOI) so that it can be cited independently in the future. For instructions see: https://journals.plos.org/plosone/s/submission-guidelines#loc-laboratory-protocols. Additionally, PLOS ONE offers an option for publishing peer-reviewed Lab Protocol articles, which describe protocols hosted on protocols.io. Read more information on sharing protocols at https://plos.org/protocols?utm_medium=editorial-email&utm_source=authorletters&utm_campaign=protocols.

As the corresponding author, your ORCID iD is verified in the submission system and will appear in the published article. PLOS supports the use of ORCID, and we encourage all coauthors to register for an ORCID iD and use it as well. Please encourage your coauthors to verify their ORCID iD within the submission system before final acceptance, as unverified ORCID iDs will not appear in the published article. *Only*  the individual author can complete the verification step; PLOS staff *cannot*  verify ORCID iDs on behalf of authors.

We look forward to receiving your revised manuscript.

Kind regards,

Ronell Bologna-Molina

Academic Editor

PLOS One

**Journal Requirements:**

1. When submitting your revision, we need you to address these additional requirements.

Please ensure that your manuscript meets PLOS ONE's style requirements, including those for file naming. The PLOS ONE style templates can be found at

https://journals.plos.org/plosone/s/file?id=wjVg/PLOSOne_formatting_sample_main_body.pdf and

https://journals.plos.org/plosone/s/file?id=ba62/PLOSOne_formatting_sample_title_authors_affiliations.pdf

2. Thank you for providing your underlying data as Supporting Information.

We note that the data set contains text or data that is not in English. Please note that PLOS is an English-language publisher, so we require data sets to be provided in English as well. Please upload an English-language version of your data set.

This will also allow us to determine if your data follows PLOS standards per our Data Availability policy here: https://journals.plos.org/plosone/s/data-availability

3. If the reviewer comments include a recommendation to cite specific previously published works, please review and evaluate these publications to determine whether they are relevant and should be cited. There is no requirement to cite these works unless the editor has indicated otherwise.

[Note: HTML markup is below. Please do not edit.]

Reviewers' comments:

Reviewer's Responses to Questions

**Comments to the Author**

1. Is the manuscript technically sound, and do the data support the conclusions?

The manuscript must describe a technically sound piece of scientific research with data that supports the conclusions. Experiments must have been conducted rigorously, with appropriate controls, replication, and sample sizes. The conclusions must be drawn appropriately based on the data presented.

Reviewer #1: Yes

Reviewer #2: No

**********

2. Has the statistical analysis been performed appropriately and rigorously?

Reviewer #1: Yes

Reviewer #2: No

**********

3. Have the authors made all data underlying the findings in their manuscript fully available?

The PLOS Data policy requires authors to make all data underlying the findings described in their manuscript fully available without restriction, with rare exception (please refer to the Data Availability Statement in the manuscript PDF file). The data should be provided as part of the manuscript or its supporting information, or deposited to a public repository. For example, in addition to summary statistics, the data points behind means, medians and variance measures should be available. If there are restrictions on publicly sharing data—e.g. participant privacy or use of data from a third party—those must be specified.

Reviewer #1: Yes

Reviewer #2: Yes

**********

4. Is the manuscript presented in an intelligible fashion and written in standard English?

PLOS ONE does not copyedit accepted manuscripts, so the language in submitted articles must be clear, correct, and unambiguous. Any typographical or grammatical errors should be corrected at revision, so please note any specific errors here.

Reviewer #1: Yes

Reviewer #2: Yes

**********

5. Review Comments to the Author

Please use the space provided to explain your answers to the questions above. You may also include additional comments for the author, including concerns about dual publication, research ethics, or publication ethics. (Please upload your review as an attachment if it exceeds 20,000 characters)

Reviewer #1: This manuscript presents a cross-sectional survey assessing adults' knowledge of oral squamous cell carcinoma (OSCC) in North-Western Italy. Using a structured, interviewer-administered questionnaire in community pharmacies (n = 914), the authors evaluate awareness levels, knowledge of risk factors and symptoms, and demographic predictors. The study also explores the potential role of community pharmacies as venues for public health education. This is a well-conducted and clearly written study that addresses an important gap in oral cancer research. The manuscript is methodologically sound, with appropriate statistical analysis and a strong discussion.

However, several areas require clarification and refinement to improve rigor and presentation. While community pharmacies provide a practical recruitment setting, they may not be representative of the general population. The discussion should be expanded to address how this sampling approach may influence health literacy levels, gender distribution, and healthcare-seeking behavior. The manuscript emphasizes pharmacies as key intervention sites; however, this conclusion was not directly tested. Statements should be reframed to reflect that pharmacies represent a potential, rather than a proven, channel for interventions. Consider using phrasing such as: “These findings suggest potential utility…” instead of making definitive claims. Although the manuscript mentions piloting of the questionnaire, it lacks sufficient detail. Additional information should be provided regarding validation procedures (e.g., reliability testing), including metrics such as Cronbach’s alpha, if available. It should also be elucidated whether the questionnaire was derived from previously validated instruments or developed specifically for this study. The use of self-reported data introduces potential recall and social desirability biases. The discussion should be expanded and further elaborated to ensure that readers understand exactly how these biases may have influenced responses, particularly regarding smoking awareness and knowledge of risk factors. The authors should explain whether non-response rates were recorded and, if so, how they were handled in the analysis. Table captions should be more detailed to ensure they are fully self-explanatory. All abbreviations (e.g., OR, CI) should be defined within the captions. Additionally, large tables (particularly Table 4) could be simplified to improve readability. Minor grammatical and stylistic issues are present throughout the manuscript. Consistent terminology should be ensured (e.g., OSCC vs. oral cancer), and typographical inconsistencies (e.g., “OCSS”) should be corrected. Some sentences in the Discussion are overly complex and could be streamlined for straightforwardness. With minor revisions to improve clarity, appropriately temper conclusions, and expand methodological detail, the manuscript would be suitable for publication. The manuscript provides meaningful insights into oral cancer awareness and highlights important public health opportunities. The abstract and introduction are generally clear and informative; however, revisions are recommended to enhance analytical depth, conceptual transparency, and overall impact. There is limited contextualization of the findings within the existing literature. The authors should strengthen their engagement with prior studies by comparing results and methodologies. Incorporating a conceptual framework would further strengthen the theoretical underpinning of the study. The conclusion should be revised to provide a more assertive interpretation of the findings while clearly emphasizing implications for public health policy and intervention strategies. Concise can be improved by reducing minor redundancies. The authors are also encouraged to provide the survey instrument and consider including supplementary materials detailing data handling procedures and variable coding to enhance reproducibility. Additional information on statistical model diagnostics would also strengthen the methodological transparency.

Reviewer #2: This manuscript addresses an important public health issue (awareness of oral squamous cell carcinoma, OSCC) and explores the role of community pharmacies as educational hubs. The study includes a relatively large sample (n=914) and applies standard statistical analyses.

However, while the topic is relevant and suitable for PLOS ONE, the manuscript requires substantial methodological clarification and reporting improvements before it can be considered for publication. The main concerns relate to questionnaire validity and reporting transparency, sampling and representativeness, statistical modeling clarity, and adherence to reporting guidelines for cross-sectional studies and survey-based research.

As this is a questionnaire-based survey, adherence to CROSS (Consensus-Based Checklist for Reporting of Survey Studies) is essential, alongside adherence to general STROBE (The Strengthening the Reporting of Observational Studies in Epidemiology) guidelines.

Major comments:

1- Questionnaire development and validation

The authors state in the manuscript that the questionnaire was piloted on a small group; however, no details are provided regarding its development or validation. No validated instrument is referenced, no psychometric evaluation is reported, and no description of item generation or expert involvement is included. This represents a critical methodological issue because the questionnaire constitutes the core of the study.

Recommendations:

The authors should provide the full questionnaire as supplementary material and include a detailed description of its development and validation. If validation was not performed, this must be explicitly acknowledged as a limitation.

2- Sampling strategy and representativeness

Participants were recruited from community pharmacies, but the sampling method is not described. The selection of pharmacies is unclear, the recruitment process is not defined, and the claim of regional representativeness is not justified. This raises concerns regarding potential selection bias and limits the generalizability of the findings.

Recommendations:

The authors should clearly define the sampling strategy, including how pharmacies and participants were selected, and avoid overgeneralization regarding representativeness.

3- Response rate and recruitment flow

No information is provided regarding the number of individuals approached, the refusal rate, or the response rate. The absence of these details limits the assessment of potential non-response bias and transparency of the recruitment process.

Recommendations:

The authors should include a participant flow diagram and report the response rate in accordance with the CROSS and STROBE recommendations.

4- Survey administration and bias

The study used interviewer-administered questionnaires, which introduces a risk of social desirability bias. However, no details are provided regarding standardization procedures or interviewer calibration. This lack of information makes it difficult to assess the reliability of data collection.

Recommendations:

The authors should describe interviewer training in more detail and explicitly acknowledge potential interviewer and social desirability bias.

5- Statistical analysis

Several concerns exist regarding the statistical approach. Variable selection based solely on univariate significance is not methodologically robust and may lead to biased models. In addition, model diagnostics are not reported in sufficient detail. Although clustering was considered in the sample size calculation, the dataset does not appear to include any clustering variable (like pharmacy identifier), and no adjustment for clustering is reported in the analysis.

Table 4 presents inconsistent reporting of regression results across outcomes. While odds ratios, confidence intervals, and p-values are fully reported for awareness of oral cancer, they are only partially reported for some outcomes (alcohol consumption and chronic trauma/irritation as risk factors) and not reported at all for others (smoking, sunlight exposure, and poor oral hygiene as risk factors). This inconsistency makes it unclear whether multivariable logistic regression analyses were performed for all outcomes, as stated in the methods, or only for selected variables.

Recommendations:

The authors should revise the statistical methods to include clinically relevant variables, report model diagnostics, and clarify whether a true cluster sampling design was implemented. If so, the lack of cluster-level data represents a methodological limitation; if not, the rationale for incorporating clustering into the sample size calculation should be explained, or the omission of clustering in the analysis justified.

The authors should ensure consistent and transparent reporting of all regression analyses, either by presenting complete models for each outcome or by clearly specifying which analyses were performed and why.

6- Outcome definition

The definition of “awareness” is unclear, as it is not specified whether it is treated as a binary variable or derived from a composite score, nor are any cut-off criteria described. This lack of clarity affects the interpretability of the results.

Recommendations:

The authors should clearly define how outcome variables were constructed and whether scoring systems were used.

7- Missing data handling

No description of missing data handling is provided, which limits transparency and reproducibility. It is clear that missing data were present (see S1 file, under column H) and, if so, how it was managed.

Recommendations:

The authors should clarify whether missing data were present and describe the methods used to address them.

8- Limitation section

The limitations section is incomplete and does not fully reflect the methodological constraints of the study. Important issues such as non-random sampling, use of a non-validated questionnaire, potential interviewer and social desirability bias, and lack of adjustment for clustering are not adequately discussed.

Recommendations:

The authors should expand this section to provide a more balanced and transparent interpretation of their findings.

9- Self-citation

A high proportion of the references cited in the manuscript appear to be authored by the same researchers in this manuscript (approximately 15 out of 44 references). While self-citation is acceptable when scientifically justified, this proportion is relatively high and may introduce bias or limit the breadth of the literature review.

Recommendations:

The authors are encouraged to ensure that the references reflect a balanced and comprehensive overview of the existing evidence, including relevant studies from independent research groups where appropriate.

Minor comments:

Correct OCSS in lines 273, 293, 332, 336, and 364 into OSCC.

Table 1 correct 22,2 into 22.2.

There is an updated version as of 2021 for reference 2.

**********

6. PLOS authors have the option to publish the peer review history of their article (what does this mean?). If published, this will include your full peer review and any attached files.

If you choose “no”, your identity will remain anonymous but your review may still be made public.

**Do you want your identity to be public for this peer review?**  For information about this choice, including consent withdrawal, please see our Privacy Policy.

Reviewer #1: No

Reviewer #2: No

**********

[NOTE: If reviewer comments were submitted as an attachment file, they will be attached to this email and accessible via the submission site. Please log into your account, locate the manuscript record, and check for the action link "View Attachments". If this link does not appear, there are no attachment files.]

To ensure your figures meet our technical requirements, please review our figure guidelines: https://journals.plos.org/plosone/s/figures

You may also use PLOS’s free figure tool, NAAS, to help you prepare publication quality figures: https://journals.plos.org/plosone/s/figures#loc-tools-for-figure-preparation.

NAAS will assess whether your figures meet our technical requirements by comparing each figure against our figure specifications.

Attachment Submitted filename: REVIEW~1.DOC

# Author comment: Author response to Decision Letter 1

**DOI:** 10.1371/journal.pone.0354509.r002

26 May 2026

Authors’ Response to the Reviewers Comments

Journal: PLOS ONE

Title of Paper: Knowledge and awareness of oral cancer among adults in North-Western Italy: a cross-sectional questionnaire-based survey in community pharmacies.

Authors: Francesca Baratta, Federica Romano, Paola Brusa, Paolo Giacomo Arduino

EDITOR:

Thank you for submitting your manuscript to PLOS ONE. After careful consideration, we feel that it has merit but does not fully meet PLOS ONE’s publication criteria as it currently stands. Therefore, we invite you to submit a revised version of the manuscript that addresses the points raised during the review process.

We sincerely thank the Editor and reviewers for their insightful comments and constructive feedback, which have significantly improved the clarity and overall quality of our manuscript. We have revised the manuscript to ensure full compliance with PLOS ONE style requirements, including file naming conventions, and we have provided an English-language version of the dataset as well as a translated version of the questionnaire as Supporting Information. We have also carefully reviewed all publications suggested by the reviewers and incorporated relevant citations into the revised manuscript where appropriate.

REVIEWER: 1

COMMENT 1:

While community pharmacies provide a practical recruitment setting, they may not be representative of the general population. The discussion should be expanded to address how this sampling approach may influence health literacy levels, gender distribution, and healthcare-seeking behavior.

Response:

Thank you for your valuable comment. We agree that recruitment via community pharmacies may limit generalizability. Pharmacy attendees are more likely to be engaged with healthcare services and may therefore have higher baseline health literacy and greater exposure to cancer awareness information than the general population. This could lead to an overestimation of cancer awareness levels and more proactive help-seeking behaviors in our sample. In addition, women may be over-represented due to their higher use of pharmacy and primary care services. These factors have been acknowledged in the Discussion, and we have clarified that the findings should be interpreted with caution when generalizing to less healthcare-engaged populations.

COMMENT 2:

The manuscript emphasizes pharmacies as key intervention sites; however, this conclusion was not directly tested. Statements should be reframed to reflect that pharmacies represent a potential, rather than a proven, channel for interventions. Consider using phrasing such as: “These findings suggest potential utility...” instead of making definitive claims.

Response:

We thank the reviewer for this helpful suggestion. We have revised the manuscript to avoid definitive statements regarding the role of community pharmacies as intervention sites and to frame pharmacies more cautiously as a potentially valuable setting for future awareness and prevention initiatives.

COMMENT 3:

Although the manuscript mentions piloting of the questionnaire, it lacks sufficient detail. Additional information should be provided regarding validation procedures (e.g., reliability testing), including metrics such as Cronbach’s alpha, if available. It should also be elucidated whether the questionnaire was derived from previously validated instruments or developed specifically for this study.

Response:

We thank the reviewer for the suggestion. More details have been added to the text to clarify how the questionnaire was developed starting from previously validated instruments. It was also explained how it was reviewed by expert reviewers and tested before being used in the study. Since the questionnaire mainly included factual and dichotomous items assessing awareness and knowledge rather than psychometric constructs, internal consistency measures such as Cronbach’s alpha were not considered appropriate.

COMMENT 4:

The use of self-reported data introduces potential recall and social desirability biases. The discussion should be expanded and further elaborated to ensure that readers understand exactly how these biases may have influenced responses, particularly regarding smoking awareness and knowledge of risk factors.

Response:

We thank the reviewer for this important comment. We agree that self-reported data may introduce recall and social desirability biases, which warrant further clarification. We have expanded the Discussion to explicitly describe how these biases may have influenced participants’ responses, particularly in relation to knowledge of oral cancer risk factors. The revised text also clarifies the potential direction of these biases, including possible overestimation of awareness and socially desirable reporting of health-related knowledge and behaviors.

COMMENT 5:

The authors should explain whether non-response rates were recorded and, if so, how they were handled in the analysis.

Response:

We thank the reviewer for this important comment. Non-response was recorded during fieldwork within each pharmacy. Eligible customers who declined participation or were unavailable were not included in the study, and recruitment proceeded consecutively until the predefined cluster targets were reached. Non-response was minimal (overall response rate: 95.1%), and the achieved sample size exceeded the minimum required sample size. Although individual-level information was not systematically collected for those who declined participation, the overall response rate was high (95.1%), which may have reduced the likelihood of substantial non-response bias. Therefore, no statistical adjustment for non-response was applied. All analyses were conducted using complete-case data from enrolled participants. This information has been added to the revised manuscript to improve transparency regarding data collection and handling procedures.

COMMENT 6:

Table captions should be more detailed to ensure they are fully self-explanatory. All abbreviations (e.g., OR, CI) should be defined within the captions. Additionally, large tables (particularly Table 4) could be simplified to improve readability.

Response:

Thank you for this valuable suggestion. We have revised all table captions to make them more detailed and self-explanatory. Specifically, all abbreviations used in the tables, including OR (Odds Ratio) and CI (Confidence Interval), are now fully defined within the respective captions. In addition, we reviewed the formatting and structure of the larger tables, particularly Table 4, and simplified their presentation to improve readability and clarity.

COMMENT 7:

Minor grammatical and stylistic issues are present throughout the manuscript. Consistent terminology should be ensured (e.g., OSCC vs. oral cancer), and typographical inconsistencies (e.g., “OCSS”) should be corrected. Some sentences in the Discussion are overly complex and could be streamlined for straightforwardness. Concise can be improved by reducing minor redundancies.

Response: We thank the reviewer for this helpful observation. We have carefully revised the manuscript to address grammatical and stylistic issues throughout the text. Consistent terminology has now been ensured, and typographical errors have been corrected. In addition, the Discussion section has been edited to improve readability by simplifying overly complex sentences and removing minor redundancies, thereby enhancing overall clarity and conciseness.

COMMENT 8:

There is limited contextualization of the findings within the existing literature. The authors should strengthen their engagement with prior studies by comparing results and methodologies. Incorporating a conceptual framework would further strengthen the theoretical underpinning of the study.

Response:

We thank the reviewer for this important comment. In the revised manuscript, we have strengthened the contextualization of our findings by comparing them with previous national and international studies investigating oral cancer awareness and related sociodemographic determinants. We have also expanded the discussion on health literacy, preventive behaviour, and healthcare engagement as factors potentially influencing awareness and healthcare-seeking attitudes related to oral cancer.

COMMENT 9:

The conclusion should be revised to provide a more assertive interpretation of the findings while clearly emphasizing implications for public health policy and intervention strategies.

Response:

We thank the reviewer for his/her valuable comment. In response, we have revised the Conclusion section to provide a more assertive interpretation of the present findings and to more clearly highlight their implications for public health policy and intervention strategies.

COMMENT 10:

The authors are also encouraged to provide the survey instrument and consider including supplementary materials detailing data handling procedures and variable coding to enhance reproducibility. Additional information on statistical model diagnostics would also strengthen the methodological transparency.

Response:

Thank you for this valuable suggestion. In response, we have provided the English versions of both the dataset and the questionnaire to improve transparency and reproducibility. We have also incorporated additional information on statistical model diagnostics in the revised manuscript to strengthen methodological clarity.

REVIEWER: 2

COMMENT 1:

Minor comments:

Correct OCSS in lines 273, 293, 332, 336, and 364 into OSCC. Table 1 correct 22,2 into 22.2.

There is an updated version as of 2021 for reference 2.

Response: Thank you for the comments. We have corrected “OCSS” to “OSCC” in lines 273, 293, 332, 336, and 364, and “22,2” to “22.2” in Table 1. Reference 2 has been updated to the 2021 version.

COMMENT 2:

Questionnaire development and validation

The authors state in the manuscript that the questionnaire was piloted on a small group; however, no details are provided regarding its development or validation. No validated instrument is referenced, no psychometric evaluation is reported, and no description of item generation or expert involvement is included. This represents a critical methodological issue because the questionnaire constitutes the core of the study.

Response:

We thank the reviewer for the suggestion. More details have been added to the text to clarify how the questionnaire was developed starting from previously validated instruments. It was also explained how it was reviewed by expert reviewers and tested before being used in the study. Since the questionnaire mainly included factual and dichotomous items assessing awareness and knowledge rather than psychometric constructs, internal consistency measures such as Cronbach’s alpha were not considered appropriate.

COMMENT 3:

Sampling strategy and representativeness

Participants were recruited from community pharmacies, but the sampling method is not described. The selection of pharmacies is unclear, the recruitment process is not defined, and the claim of regional representativeness is not justified. This raises concerns regarding potential selection bias and limits the generalizability of the findings.

Response:

We thank the reviewer for this helpful observation. The manuscript has been carefully revised to detail how pharmacy recruitment was conducted to ensure the variability across provinces and urban-rural settings.

COMMENT 4:

Response rate and recruitment flow

No information is provided regarding the number of individuals approached, the refusal rate, or the response rate. The absence of these details limits the assessment of potential non-response bias and transparency of the recruitment process.

Response:

Thank you for the comment. We have revised the Methods and Results sections to provide additional details on the recruitment flow. Eligible pharmacy customers were consecutively approached, and 914 out of 961 agreed to participate, yielding an overall response rate of 95.1%. Although individual-level information was not systematically collected for those who declined participation, the high response rate may have reduced the likelihood of substantial non-response bias. This limitation has been acknowledged in the revised manuscript.

COMMENT 5:

Survey administration and bias

The study used interviewer-administered questionnaires, which introduces a risk of social desirability bias. However, no details are provided regarding standardization procedures or interviewer calibration. This lack of information makes it difficult to assess the reliability of data collection.

Response:

Thank you for this important comment. We would like to clarify that all interviewers underwent prior calibration and were trained using standardized procedures before data collection. This included guidance on consistent question delivery and minimizing interviewer influence. We will revise the manuscript to explicitly describe these steps to improve transparency and allow better assessment of the reliability of data collection.

COMMENT 6:

Statistical analysis

Several concerns exist regarding the statistical approach. Variable selection based solely on univariate significance is not methodologically robust and may lead to biased models. In addition, model diagnostics are not reported in sufficient detail. Although clustering was considered in the sample size calculation, the dataset does not appear to include any clustering variable (like pharmacy identifier), and no adjustment for clustering is reported in the analysis. Table 4 presents inconsistent reporting of regression results across outcomes. While odds ratios, confidence intervals, and p-values are fully reported for awareness of oral cancer, they are only partially reported for some outcomes (alcohol consumption and chronic trauma/irritation as risk factors) and not reported at all for others (smoking, sunlight exposure, and poor oral hygiene as risk factors). This inconsistency makes it unclear whether multivariable logistic regression analyses were performed for all outcomes, as stated in the methods, or only for selected variables.

Response:

Thank you for these important comments. We revised the manuscript to improve the clarity and robustness of the statistical analysis. Variable selection for multivariable logistic regression is now described as being based on both theoretical relevance and prior literature, rather than solely on univariate significance. Additional information on goodness-of-fit assessment has also been added.

We further clarified that clustering was considered during sample size calculation; however, pharmacy identifiers were not retained in the anonymized dataset, preventing clustering adjustment in the final analyses. This limitation is now acknowledged in the manuscript. However, given the relatively large number of participating pharmacies and the exploratory nature of the study, the impact on the estimated associations is expected to be limited. Finally, Table 4 has been revised to ensure consistent reporting across all outcomes. Odds ratios (ORs), 95% confidence intervals (CIs), and p-values are now uniformly presented for all multivariable logistic regression analyses, and the Methods and Results sections clearly state that these analyses were performed for all specified outcomes.

COMMENT 7:

Outcome definition

The definition of “awareness” is unclear, as it is not specified whether it is treated as a binary variable or derived from a composite score, nor are any cut-off criteria described. This lack of clarity affects the interpretability of the results.

Response:

Thank you for highlighting this point. We agree that the definition of “awareness” required clarification. In our study, awareness was assessed using a single item from the questionnaire and treated as a binary variable. Specifically, participants were classified as “aware” if they responded “yes” to the question whether they had previously heard of oral cancer, and “not aware” if they responded “no.” No composite score or cut-off criteria were applied. We have now clarified this definition in the Methods section to improve transparency and interpretability.

COMMENT 8:

Missing data han

Attachment Submitted filename: Response Letter.pdf

# Peer review: Decision Letter 1

**DOI:** 10.1371/journal.pone.0354509.r003

**Authors:** Ronell Bologna-Molina

16 Jun 2026

PONE-D-26-13277R1

Knowledge and awareness of oral cancer among adults in North-Western Italy: a cross-sectional questionnaire-based survey in community pharmacies.

PLOS One

Dear Dr. Romano,

Thank you for submitting your manuscript to PLOS ONE. After careful consideration, we feel that it has merit but does not fully meet PLOS ONE’s publication criteria as it currently stands. Therefore, we invite you to submit a revised version of the manuscript that addresses the points raised during the review process.

==============================

The manuscript is considered scientifically sound and has improved considerably following revision. The authors are requested to address a few minor issues related to reporting transparency, methodological limitations, and reference selection.  Please refer to the reviewer’s comments below for specific recommendations.

==============================

Please submit your revised manuscript by Jul 31 2026 11:59PM. If you will need more time than this to complete your revisions, please reply to this message or contact the journal office at plosone@plos.org. When you're ready to submit your revision, log on to https://www.editorialmanager.com/pone/ and select the 'Submissions Needing Revision' folder to locate your manuscript file.

Please include the following items when submitting your revised manuscript:

- A letter that responds to each point raised by the academic editor and reviewer(s). You should upload this letter as a separate file labeled 'Response to Reviewers'.
- A marked-up copy of your manuscript that highlights changes made to the original version. You should upload this as a separate file labeled 'Revised Manuscript with Track Changes'.
- An unmarked version of your revised paper without tracked changes. You should upload this as a separate file labeled 'Manuscript'.

If you would like to make changes to your financial disclosure, please include your updated statement in your cover letter. Guidelines for resubmitting your figure files are available below the reviewer comments at the end of this letter.

If applicable, we recommend that you deposit your laboratory protocols in protocols.io to enhance the reproducibility of your results. Protocols.io assigns your protocol its own identifier (DOI) so that it can be cited independently in the future. For instructions see: https://journals.plos.org/plosone/s/submission-guidelines#loc-laboratory-protocols. Additionally, PLOS ONE offers an option for publishing peer-reviewed Lab Protocol articles, which describe protocols hosted on protocols.io. Read more information on sharing protocols at https://plos.org/protocols?utm_medium=editorial-email&utm_source=authorletters&utm_campaign=protocols.

As the corresponding author, your ORCID iD is verified in the submission system and will appear in the published article. PLOS supports the use of ORCID, and we encourage all coauthors to register for an ORCID iD and use it as well. Please encourage your coauthors to verify their ORCID iD within the submission system before final acceptance, as unverified ORCID iDs will not appear in the published article. *Only*  the individual author can complete the verification step; PLOS staff *cannot*  verify ORCID iDs on behalf of authors.

We look forward to receiving your revised manuscript.

Kind regards,

Ronell Bologna-Molina

Academic Editor

PLOS One

Journal Requirements:

If the reviewer comments include a recommendation to cite specific previously published works, please review and evaluate these publications to determine whether they are relevant and should be cited. There is no requirement to cite these works unless the editor has indicated otherwise.

Please review your reference list to ensure that it is complete and correct. If you have cited papers that have been retracted, please include the rationale for doing so in the manuscript text, or remove these references and replace them with relevant current references. Any changes to the reference list should be mentioned in the rebuttal letter that accompanies your revised manuscript. If you need to cite a retracted article, indicate the article’s retracted status in the References list and also include a citation and full reference for the retraction notice.

Additional Editor Comments:

The manuscript is considered scientifically sound and has improved considerably following revision. The authors are requested to address a few minor issues related to reporting transparency, methodological limitations, and reference selection. These points do not affect the overall conclusions of the study but should be considered to further enhance the clarity and rigor of the manuscript. Please refer to the reviewer’s comments below for specific recommendations.

[Note: HTML markup is below. Please do not edit.]

Reviewers' comments:

Reviewer's Responses to Questions

**Comments to the Author**

1. If the authors have adequately addressed your comments raised in a previous round of review and you feel that this manuscript is now acceptable for publication, you may indicate that here to bypass the “Comments to the Author” section, enter your conflict of interest statement in the “Confidential to Editor” section, and submit your "Accept" recommendation.

Reviewer #1: All comments have been addressed

Reviewer #2: (No Response)

**********

2. Is the manuscript technically sound, and do the data support the conclusions?

The manuscript must describe a technically sound piece of scientific research with data that supports the conclusions. Experiments must have been conducted rigorously, with appropriate controls, replication, and sample sizes. The conclusions must be drawn appropriately based on the data presented.

Reviewer #1: Yes

Reviewer #2: Partly

**********

3. Has the statistical analysis been performed appropriately and rigorously?

Reviewer #1: Yes

Reviewer #2: Yes

**********

4. Have the authors made all data underlying the findings in their manuscript fully available?

The PLOS Data policy requires authors to make all data underlying the findings described in their manuscript fully available without restriction, with rare exception (please refer to the Data Availability Statement in the manuscript PDF file). The data should be provided as part of the manuscript or its supporting information, or deposited to a public repository. For example, in addition to summary statistics, the data points behind means, medians and variance measures should be available. If there are restrictions on publicly sharing data—e.g. participant privacy or use of data from a third party—those must be specified.

Reviewer #1: Yes

Reviewer #2: Yes

**********

5. Is the manuscript presented in an intelligible fashion and written in standard English?

PLOS ONE does not copyedit accepted manuscripts, so the language in submitted articles must be clear, correct, and unambiguous. Any typographical or grammatical errors should be corrected at revision, so please note any specific errors here.

Reviewer #1: Yes

Reviewer #2: Yes

**********

6. Review Comments to the Author

Please use the space provided to explain your answers to the questions above. You may also include additional comments for the author, including concerns about dual publication, research ethics, or publication ethics. (Please upload your review as an attachment if it exceeds 20,000 characters)

Reviewer #1: The authors investigate public knowledge and awareness of Oral Squamous Cell Carcinoma (OSCC) and its associated risk factors among adults in Northwest Italy. Using a cross-sectional survey of 914 participants recruited through community pharmacies, the study demonstrates that although 70.4% of respondents were aware of OSCC, important knowledge gaps remain regarding its aggressiveness and less-recognized risk factors, including alcohol consumption and sun exposure. The findings identify gender, educational level, and smoking status as significant predictors of awareness.

The manuscript is well-grounded in the existing literature. The authors appropriately contextualize their findings by comparing them with previous Italian and European studies. While national-level evidence exists, regional data remain fragmented; therefore, the study provides an insight into a specific geographic area and highlights the potential role of community pharmacies as settings for public health initiatives.

The presentation of results is clear and effective. Tables 1–6 are logically organized and directly support the study findings. In response to previous reviewer comments, table captions have been expanded, and abbreviations such as OR (Odds Ratio) and CI (Confidence Interval) are clearly defined. The use of frequencies and adjusted odds ratios (AORs) is appropriate for the cross-sectional design and analytical objectives.

The methodology is soundly suited to the research question. The face-to-face, observational cross-sectional survey design is appropriate for assessing awareness and knowledge within the target population. The sample size was adequately calculated (target n = 910; achieved n = 914), and potential confounding factors were addressed through multivariable logistic regression analyses. The inclusion of the full dataset (S3 File) and the translated questionnaire (S1 File) enhances transparency and reproducibility.

The authors provide a balanced discussion of the study limitations. They acknowledge the possibility of selection bias arising from pharmacy-based recruitment, as participants may be more health-conscious than the general population. Potential recall and social desirability biases inherent to face-to-face interviews are also appropriately discussed. Furthermore, the authors transparently explain that although clustering was considered during sample size estimation, cluster-adjusted analyses could not be performed because pharmacy identifiers were removed during anonymization.

The study adheres to ethical standards, having received approval from the Bioethics Committee of the University of Turin (Protocol No. 0494208), and written informed consent was obtained from all participants. Reporting follows STROBE guidelines for observational studies.

Importantly, the conclusions are supported by the results. The authors have revised earlier overstatements and now appropriately describe community pharmacies as a potential, rather than proven, setting for future interventions. The finding that 92.2% of respondents expressed a desire for additional information and 67.3% identified pharmacies as their preferred information source provides practical guidance for future awareness campaigns and public health strategies.

Overall, the manuscript is well-structured, methodologically rigorous, transparent in its reporting, and responsive to previous reviewers' concerns. It makes a meaningful contribution to the literature on OSCC awareness and offers actionable insights for public health practice.

Reviewer #2: 1- Reporting guidelines adherence:

Although the revised manuscript references the STROBE guideline and reporting quality has substantially improved, formal adherence to EQUATOR reporting standards remains incomplete. In particular, a completed STROBE checklist was not provided, and alignment with CROSS remains limited.

Recommendation:

The authors are encouraged to submit a completed STROBE checklist and improve explicit alignment with CROSS recommendations to further strengthen methodological transparency and reporting rigor. PLOS ONE instructions are clear: Methodological integrity and transparency come first.

2- Questionnaire development and validation

The manuscript still lacks sufficient information regarding item adaptation from previous instruments and does not include full psychometric validation procedures.

Recommendation:

The authors should expand the limitation section to include that the questionnaire did not undergo full psychometric validation.

3- Clustering and statistical analysis

The potential effect of unadjusted clustering on estimate precision remains important.

Recommendation:

The limitation regarding the lack of clustering adjustment should be stated more cautiously, specifically acknowledging that standard errors and confidence intervals may potentially be underestimated.

4- Self-Citation and number of references:

Instead of removing references, replace or increase them. Self-cited references have been reduced, but are still high unless scientifically justified.

Overall assessment:

The revised manuscript has improved substantially compared with the original submission, and most previous methodological and reporting concerns were adequately addressed. The study addresses an important public health topic and provides useful regional data regarding oral cancer awareness.

Adherence to Equator reporting guidelines and instructions is the major concern.

**********

7. PLOS authors have the option to publish the peer review history of their article (what does this mean?). If published, this will include your full peer review and any attached files.

If you choose “no”, your identity will remain anonymous but your review may still be made public.

**Do you want your identity to be public for this peer review?**  For information about this choice, including consent withdrawal, please see our Privacy Policy.

Reviewer #1: No

Reviewer #2: **Yes:**  Gamil Al-Madhagy

**********

[NOTE: If reviewer comments were submitted as an attachment file, they will be attached to this email and accessible via the submission site. Please log into your account, locate the manuscript record, and check for the action link "View Attachments". If this link does not appear, there are no attachment files.]

To ensure your figures meet our technical requirements, please review our figure guidelines: https://journals.plos.org/plosone/s/figures

You may also use PLOS’s free figure tool, NAAS, to help you prepare publication quality figures: https://journals.plos.org/plosone/s/figures#loc-tools-for-figure-preparation.

NAAS will assess whether your figures meet our technical requirements by comparing each figure against our figure specifications.

Attachment Submitted filename: REVIEWER CONSIDERATIONS.pdf

# Author comment: Author response to Decision Letter 2

**DOI:** 10.1371/journal.pone.0354509.r004

8 Jul 2026

EDITOR:

The manuscript is considered scientifically sound and has improved considerably following revision. The authors are requested to address a few minor issues related to reporting transparency, methodological limitations, and reference selection. These points do not affect the overall conclusions of the study but should be considered to further enhance the clarity and rigor of the manuscript. Please refer to the reviewer’s comments below for specific recommendations.

We sincerely thank the Editor and reviewers for the positive evaluation of our manuscript and for recognizing the substantial improvements made during the revision process. We have carefully considered all comments and have revised the manuscript accordingly. Specifically, we have strengthened adherence to reporting guidelines, expanded the discussion of methodological limitations, clarified aspects of questionnaire development, and further reviewed the reference list to ensure a balanced and comprehensive representation of the relevant literature.

REVIEWER: 1

COMMENT: The authors investigate public knowledge and awareness of Oral Squamous Cell Carcinoma (OSCC) and its associated risk factors among adults in Northwest Italy. Using a cross-sectional survey of 914 participants recruited through community pharmacies, the study demonstrates that although 70.4% of respondents were aware of OSCC, important knowledge gaps remain regarding its aggressiveness and less-recognized risk factors, including alcohol consumption and sun exposure. The findings identify gender, educational level, and smoking status as significant predictors of awareness.

The manuscript is well-grounded in the existing literature. The authors appropriately contextualize their findings by comparing them with previous Italian and European studies. While national-level evidence exists, regional data remain fragmented; therefore, the study provides an insight into a specific geographic area and highlights the potential role of community pharmacies as settings for public health initiatives.

The presentation of results is clear and effective. Tables 1–6 are logically organized and directly support the study findings. In response to previous reviewer comments, table captions have been expanded, and abbreviations such as OR (Odds Ratio) and CI (Confidence Interval) are clearly defined. The use of frequencies and adjusted odds ratios (AORs) is appropriate for the cross-sectional design and analytical objectives.

The methodology is soundly suited to the research question. The face-to-face, observational cross-sectional survey design is appropriate for assessing awareness and knowledge within the target population. The sample size was adequately calculated (target n = 910; achieved n = 914), and potential confounding factors were addressed through multivariable logistic regression analyses. The inclusion of the full dataset (S3 File) and the translated questionnaire (S1 File) enhances transparency and reproducibility.

The authors provide a balanced discussion of the study limitations. They acknowledge the possibility of selection bias arising from pharmacy-based recruitment, as participants may be more health-conscious than the general population. Potential recall and social desirability biases inherent to face-to-face interviews are also appropriately discussed. Furthermore, the authors transparently explain that although clustering was considered during sample size estimation, cluster-adjusted analyses could not be performed because pharmacy identifiers were removed during anonymization. The study adheres to ethical standards, having received approval from the Bioethics Committee of the University of Turin (Protocol No. 0494208), and written informed consent was obtained from all participants. Reporting follows STROBE guidelines for observational studies.

Importantly, the conclusions are supported by the results. The authors have revised earlier overstatements and now appropriately describe community pharmacies as a potential, rather than proven, setting for future interventions. The finding that 92.2% of respondents expressed a desire for additional information and 67.3% identified pharmacies as their preferred information source provides practical guidance for future awareness campaigns and public health strategies.

Overall, the manuscript is well-structured, methodologically rigorous, transparent in its reporting, and responsive to previous reviewers' concerns. It makes a meaningful contribution to the literature on OSCC awareness and offers actionable insights for public health practice.

Response:

We sincerely thank the reviewer for the positive and constructive evaluation of our manuscript. We are pleased that the reviewer considers the study a meaningful contribution to the literature on oral squamous cell carcinoma awareness and recognizes the potential public health implications of our findings. The reviewer's constructive comments and recommendations have strengthened the clarity and quality of the manuscript.

REVIEWER: 2

COMMENT 1:

Although the revised manuscript references the STROBE guideline and reporting quality has substantially improved, formal adherence to EQUATOR reporting standards remains incomplete. In particular, a completed STROBE checklist was not provided, and alignment with CROSS remains limited.

Recommendation:

The authors are encouraged to submit a completed STROBE checklist and improve explicit alignment with CROSS recommendations to further strengthen methodological transparency and reporting rigor. PLOS ONE instructions are clear: Methodological integrity and transparency come first.

Response: We appreciate this important observation. In response, we have prepared and submitted a completed STROBE checklist as supplementary material, with page references indicating where each reporting item is addressed in the manuscript. We have also reviewed the manuscript against the CROSS recommendations and further strengthened the reporting of study design, sampling procedures, questionnaire administration, and statistical methods. These revisions have enhanced the methodological transparency and reporting rigor in accordance with EQUATOR Network guidance and PLOS ONE reporting requirements.

COMMENT 2:

Questionnaire development and validation

The manuscript still lacks sufficient information regarding item adaptation from previous instruments and does not include full psychometric validation procedures.

Recommendation:

The authors should expand the limitation section to include that the questionnaire did not undergo full psychometric validation.

Response:

We thank the reviewer for this valuable comment. We have expanded the description of questionnaire development in the Methods section, including clarification of the sources from which questionnaire items were adapted and the process used for content review and refinement. We agree that the questionnaire did not undergo a complete psychometric validation process. Therefore, we have explicitly acknowledged this limitation in the revised manuscript.

COMMENT 3:

Clustering and statistical analysis

The potential effect of unadjusted clustering on estimate precision remains important.

Recommendation:

The limitation regarding the lack of clustering adjustment should be stated more cautiously, specifically acknowledging that standard errors and confidence intervals may potentially be underestimated.

Response:

We appreciate this observation and agree that this issue warrants clearer discussion. Accordingly, we have revised the Limitations section to explicitly acknowledge that clustering effects were not formally accounted for in the analysis. We now state that the absence of clustering adjustment may have resulted in underestimated standard errors and confidence intervals, potentially affecting the precision of some estimates. This limitation has been added to ensure a more balanced interpretation of the study findings.

COMMENT 4:

Self-Citation and number of references:

Instead of removing references, replace or increase them. Self-cited references have been reduced, but are still high unless scientifically justified.

Response:

We thank the reviewer for this suggestion. We carefully re-evaluated the reference list and incorporated additional relevant references to provide a more comprehensive contextual framework. Self-citations were reviewed individually and retained only when directly relevant to the study objectives, methodology, or interpretation of findings. We believe the revised reference list now achieves a more balanced representation of the available literature while maintaining appropriate citation of prior work that is specifically pertinent to the present study.

Attachment Submitted filename: Response Letter.docx

# Editor report: Decision Letter 2

**DOI:** 10.1371/journal.pone.0354509.r005

**Authors:** Ronell Bologna-Molina

10 Jul 2026

Knowledge and awareness of oral cancer among adults in North-Western Italy: a cross-sectional questionnaire-based survey in community pharmacies.

PONE-D-26-13277R2

Dear Dr. Romano,

We’re pleased to inform you that your manuscript has been judged scientifically suitable for publication and will be formally accepted for publication once it meets all outstanding technical requirements.

Within one week, you’ll receive an e-mail detailing the required amendments. When these have been addressed, you’ll receive a formal acceptance letter and your manuscript will be scheduled for publication.

An invoice will be generated when your article is formally accepted. Please note, if your institution has a publishing partnership with PLOS and your article meets the relevant criteria, all or part of your publication costs will be covered. Please make sure your user information is up-to-date by logging into Editorial Manager at Editorial Manager® and clicking the ‘Update My Information' link at the top of the page. For questions related to billing, please contact billing support.

If your institution or institutions have a press office, please notify them about your upcoming paper to help maximize its impact. If they’ll be preparing press materials, please inform our press team as soon as possible -- no later than 48 hours after receiving the formal acceptance. Your manuscript will remain under strict press embargo until 2 pm Eastern Time on the date of publication. For more information, please contact onepress@plos.org.

Kind regards,

Ronell Bologna-Molina

Academic Editor

PLOS One

Additional Editor Comments (optional):

The manuscript has been substantially improved and is now suitable for publication. The authors have adequately addressed the reviewers' comments and implemented the requested revisions.

Reviewers' comments:

# Editor report: Acceptance letter

**DOI:** 10.1371/journal.pone.0354509.r006

**Authors:** Ronell Bologna-Molina

PONE-D-26-13277R2

PLOS One

Dear Dr. Romano,

I'm pleased to inform you that your manuscript has been deemed suitable for publication in PLOS One. Congratulations! Your manuscript is now being handed over to our production team.

At this stage, our production department will prepare your paper for publication. This includes ensuring the following:

* All references, tables, and figures are properly cited

* All relevant supporting information is included in the manuscript submission,

* There are no issues that prevent the paper from being properly typeset

You will receive further instructions from the production team, including instructions on how to review your proof when it is ready. Please keep in mind that we are working through a large volume of accepted articles, so please give us a few days to review your paper and let you know the next and final steps.

Lastly, if your institution or institutions have a press office, please let them know about your upcoming paper now to help maximize its impact. If they'll be preparing press materials, please inform our press team within the next 48 hours. Your manuscript will remain under strict press embargo until 2 pm Eastern Time on the date of publication. For more information, please contact onepress@plos.org.

You will receive an invoice from PLOS for your publication fee after your manuscript has reached the completed accept phase. If you receive an email requesting payment before acceptance or for any other service, this may be a phishing scheme. Learn how to identify phishing emails and protect your accounts at https://explore.plos.org/phishing.

If we can help with anything else, please email us at customercare@plos.org.

Thank you for submitting your work to PLOS ONE and supporting open access.

Kind regards,

PLOS ONE Editorial Office Staff

on behalf of

Professor Ronell Bologna-Molina

Academic Editor

PLOS One
